# A comparative study on deep learning models for text classification of unstructured medical notes with various levels of class imbalance

**DOI:** 10.1186/s12874-022-01665-y

**Published:** 2022-07-02

**Authors:** Hongxia Lu, Louis Ehwerhemuepha, Cyril Rakovski

**Affiliations:** 1 Schmid College of Science and Technology, Chapman University, 1 University Dr, Orange, CA 92866 USA; 2 Children’s Health of Orange County (CHOC), Orange, CA 92868 USA

**Keywords:** Medical notes, Text classification, BERT, CNN, Deep learning, Embedding, Transformer encoder

## Abstract

**Background:**

Discharge medical notes written by physicians contain important information about the health condition of patients. Many deep learning algorithms have been successfully applied to extract important information from unstructured medical notes data that can entail subsequent actionable results in the medical domain. This study aims to explore the model performance of various deep learning algorithms in text classification tasks on medical notes with respect to different disease class imbalance scenarios.

**Methods:**

In this study, we employed seven artificial intelligence models, a CNN (Convolutional Neural Network), a Transformer encoder, a pretrained BERT (Bidirectional Encoder Representations from Transformers), and four typical sequence neural networks models, namely, RNN (Recurrent Neural Network), GRU (Gated Recurrent Unit), LSTM (Long Short-Term Memory), and Bi-LSTM (Bi-directional Long Short-Term Memory) to classify the presence or absence of 16 disease conditions from patients’ discharge summary notes. We analyzed this question as a composition of 16 binary separate classification problems. The model performance of the seven models on each of the 16 datasets with various levels of imbalance between classes were compared in terms of AUC-ROC (Area Under the Curve of the Receiver Operating Characteristic), AUC-PR (Area Under the Curve of Precision and Recall), F1 Score, and Balanced Accuracy as well as the training time. The model performances were also compared in combination with different word embedding approaches (GloVe, BioWordVec, and no pre-trained word embeddings).

**Results:**

The analyses of these 16 binary classification problems showed that the Transformer encoder model performs the best in nearly all scenarios. In addition, when the disease prevalence is close to or greater than 50%, the Convolutional Neural Network model achieved a comparable performance to the Transformer encoder, and its training time was 17.6% shorter than the second fastest model, 91.3% shorter than the Transformer encoder, and 94.7% shorter than the pre-trained BERT-Base model. The BioWordVec embeddings slightly improved the performance of the Bi-LSTM model in most disease prevalence scenarios, while the CNN model performed better without pre-trained word embeddings. In addition, the training time was significantly reduced with the GloVe embeddings for all models.

**Conclusions:**

For classification tasks on medical notes, Transformer encoders are the best choice if the computation resource is not an issue. Otherwise, when the classes are relatively balanced, CNNs are a leading candidate because of their competitive performance and computational efficiency.

**Supplementary Information:**

The online version contains supplementary material available at 10.1186/s12874-022-01665-y.

## Background

Unstructured medical notes such as discharge summaries are valuable health records that contain rich clinical information about patients’ health conditions. Some of the disease details may not be reflected in the structured data fields. Many studies have been carried out to extract additional information from unstructured medical notes and make mortality predictions based on these data alone [1–4]. This study aims to explore the model performance of various deep learning algorithms in text classification tasks on medical notes to help point the attention of the research community to the potentials of text classification and the behaviors of various NLP (Natural Language Processing) algorithms on medical notes data in different class imbalance scenarios. The algorithms compared in this study include traditional recurrence networks such as RNN, GRU, LSTM, Bi-LSTM, as well as CNN, and attention algorithms such as the Transformer encoder and BERT-Base. The model performances were evaluated in terms of AUC-ROC, AUC-PR, F1 Score, and Balanced Accuracy. Deep learning algorithms such as RNN, GRU, LSTM, and Bi-LSTM models are typically used for NLP tasks and have achieved promising results [5–7]. They are designed to work with sequence data by allowing previous outputs to be used as inputs which allows flow of information from previous elements (and posterior elements in Bi-LSTMs) of the sequence.

GRUs, LSTMs, and Bi-LSTMs are advanced variants of the vanilla RNNs with additional gates (mathematical operations involving additional weights to be trained) added in an RNN unit to overcome the vanishing or exploding gradient problem that RNNs often suffer with long sequences. LSTMs have two additional gates compared to GRUs which entails a better performance on long sequences by allowing information from further back to be carried over to the current unit. Bi-LSTMs have an additional layer (on top of the LSTM units) that goes backwards so that the information from posterior elements is passed on to previous units. This feature works particularly well for text data since the context (information from both previous elements and from posterior elements) is important for interpretation of words. The extra gates and layer, however, consequently result in more complexity and longer training time.

More recently, CNNs have attracted attention for NLP tasks due to their superior performance especially on lengthy texts [8–11]. CNNs are widely used in computer vision such as image classification or image recognition [12, 13]. CNNs in computer vision feature a 2-Dimensional or 3-Dimensional convolutional layer that extracts information from neighboring pixels and thus recognizes patterns across space. CNNs in NLP tasks employ a 1-Dimensional convolutional layer which extracts information from adjacent words. It is not quite clear exactly why CNN outperforms the traditional NLP algorithms such as RNN, GRU, LSTM, and Bi-LSTM in many cases but it is widely accepted that the number of kernels in the 1-dimensional convolutional layer in CNN serves as the n-gram (n adjacent words treated as one) technique in NLP [14–16]. In traditional NLP algorithms, anything more than 3-g would be too cumbersome. CNN algorithms, however, can easily adopt an 8-g or even higher gram technique (depending on the length of the text) without increasing the computational cost.

Transformers have been successfully applied to many NLP tasks since the introduction of the Transformer by Vaswani et al. [17]. The Transformer model is a novel network architecture that is based solely on attention mechanisms, dispensing with recurrence and convolutions entirely [17]. Transformers for tasks such as translation or question answering have both encoders and decoders, while Transformers for text classification tasks typically have only encoders. An encoder has two layers, a multi-head self-attention layer and a feedforward layer. Unlike the recurrent networks which process the words sequentially by taking the information from the previous word as input for the processing of the current word, the Transformer processes an input sequence as a whole. Another novel design of the Transformer is that it introduces positional embedding which captures information from the order of words. The positional embeddings are added to the word embeddings before they are fed to the encoder. One major disadvantage of Transformers is their high computational cost especially when the text sequences are long. Longer sequences are disproportionately expensive because attention is quadratic to the sequence length due to the self-attention of each word with every other word in the sequence [18].

BERT (Bi-directional Encoder Representations from Transformers) is a transformer-based language representation model which was designed to pre-train deep bidirectional representations from unlabeled text from BooksCorpus and English Wikipedia [18]. Two architectures of the BERT model (BERT-Base and BERT-Large) were introduced in the original paper. The BERT-Base model has 12 Transformer encoders, 12 self-attention heads in each encoder, a hidden size of 768, and a total of 110 M parameters. The BERT-Large model has 24 Transformer encoders, 16 self-attention heads in each encoder, a hidden size of 1024, and a total of 340 M parameters. The BERT model achieved state-of-the-art performance on a number of natural language understanding tasks when it was published. It has been successfully applied in many NLP tasks since then [19–21]. One major drawback of BERT is the costly computational resources needed to train or fine-tune the model due to the large number of parameters [22].

### Data

In this study, we used de-identified discharge summary data made available by Harvard University in 2008 for a challenge to classify obesity and its comorbidities with multiple classes (presence, absence, or questionable) for each disease that were annotated with textual judgments and intuitive judgments, respectively [23]. The data consist of 1,237 unique discharge summaries from the Partners HealthCare Research Patient Data Repository and were annotated from a list of 16 disease conditions by three experts from the Massachusetts General Hospital [24]. The literature on classification tasks using this dataset is focused on optimizing the macro-F score of the multi-class classification task by primarily employing rule-based methods (or rule-based methods combined with traditional machine learning algorithms such as SVM) which involved heavy text preprocessing that are tailored for these specific discharge summaries in association with these 16 diseases [24]. For example, Ware et al. employed the Apelon terminology engine to provide synonym sets for drug names and used Domain Specific Language (DSL) to frame the rules to identify the presence of a disease [25]. Yang et al. built a dictionary for diseases, symptoms, treatments, medications, and their synonyms [26]. Solt et al. also developed a regular expression driven string replacement dictionary for all occurrences of relevant abbreviations, synonyms, plain English equivalents, spelling variants, frequent typos, suffixed forms, etc. [27].

The goal of this study is to compare the behavior of the 7 deep learning algorithms in terms of their performance on the same datasets, their training efficiency, and their ability to handle imbalanced classes, as well as the effect of two types of word embedding approaches. Therefore, we simplified the multi-class task into a binary-class problem for the intuitive labels only and applied general text preprocessing. We converted the data into 16 datasets for binary classifications, each with the same 1,237 discharge summaries but a different binary outcome variable denoting the presence or absence of a particular disease. The disease prevalence of the 16 disease conditions in the datasets are listed in Table 1. The disease prevalence ranges from 5 to 73%, with hypertriglyceridemia being the least prevalent and hypertension the most prevalent. The disease prevalence also reflects the class imbalance level between the positive (disease presence) and negative (disease absence) classes in our binary classification problems.

**Table 1:** Disease prevalence (*N* = 1,237)

Disease	Disease Prevalence	Disease	Disease Prevalence
Hypertriglyceridemia	5%	GERD*	20%
Venous Insufficiency	7%	Depression	20%
Asthma	13%	Obesity	40%
Gout	13%	CHF*	43%
OSA*	14%	Hypercholesterolemia	47%
PVD*	15%	CAD*	55%
Gallstones	15%	Diabetes	66%
OA*	18%	Hypertension	73%

OSA* obstructive sleep apnea, PVD* peripheral vascular disease , OA* osteo arthritis, GERD* gastroesophageal reflux disease, CHF* congestive heart failure, CAD* coronary artery disease

The discharge summary notes in the dataset include contents such as the description of the current illness, medical history, information about physical examination and laboratory examination, treatment or services provided if applicable, and discharge medications. These unstructured medical notes require special treatment before they can be fed into deep learning algorithms. We first converted all words to the lower case so that words such as “disease” and “Disease” are treated as the same word. We then removed numbers and punctuations which do not carry significant information about the diagnoses. Standard stop words (the most common words in any natural language which do not add much value in NLP modeling) such as “the”, “this”, “that” were removed as well as template words such as “discharge”, “admission”, “date”, and words with only one or two characters such as “mg”. Detailed descriptive statistics of the variables denoting the number of words and characters in the discharge summaries before and after cleaning are shown in Table 2. In particular, the average number of words before and after cleaning were 1170 and 557 with a minimum of 146 and 50 and a maximum of 4280 and 2098, respectively. Similarly, the average number of characters before and after cleaning were 6870 and 4429 with a minimum of 903 and 410 and a maximum of 25,842 and 16,976, respectively.

**Table 2:** Descriptive statistics

Descriptive Statistics	Number of Words		Number of Characters	
	**Before Cleaning**	**After Cleaning**	**Before Cleaning**	**After Cleaning**
Minimum	146	50	903	410
25% Percentile	819	391	4798	3089
Median	1084	517	6391	4098
Mean	1170	557	6870	4429
75% Percentile	1425	687	8404	5420
Maximum	4280	2098	25,842	16,976
Standard Deviation	506	242	2960	1931

The dataset was then randomly split (stratified according to the disease label) into training and test sets containing 75% and 25% of the data, respectively, for 10 iterations and the average metrics from the 10 iterations were used for comparison of the model performance. In each iteration, the dataset was randomly split with stratification and the models were trained on the same training set and tested on the same test set. Regular tokenizing with Keras Tokenizer was performed to convert text into numbers for all models except for BERT which uses a different tokenization technique (WordPiece tokenization) [18, 28]. The regular tokenizing procedure takes the following two steps. First, it creates a word-index dictionary based on word frequency in the training set so that every unique word is assigned an integer value as the index (an integer between 1 and the maximum number of unique words in the texts. 0 is reserved for padding.) Then, it transforms each text to a sequence of integers by taking each word in the discharge summary note, looking it up in the word-index dictionary, and replacing it with its corresponding index. Next, the medical notes in the test set were converted to sequences of integers by looking up each word in the word-index dictionary previously constructed from the training set. The reason that the word-index dictionary is built based on the training set only is to avoid information leaking from the test set, because the test set is supposed to contain new data that the model has never seen. At this point, all medical notes have been converted to numbers but they are of different lengths because each discharge note has different length. We arbitrarily chose the maximum sequence length to be 557 (the average length of the sequences in the dataset) and forced all sequences to be of the same length by truncating the longer sequences and padding the shorter sequences with 0’s. After these preprocessing steps, the original discharge summary notes have been transformed into sequences of integers of the same length and ready to be fed into the deep learning models. For the pre-trained BERT-Base model, the maximum sequence length allowed is 512.

## Methods

A CNN model with eight 1-dimensional filters and a kernel size of eight, a RNN model with eight units, a GRU model with eight units, a LSTM model with eight units, a Bi-LSTM model with eight units, and a Transformer encoder with one encoder and two self-attention heads were fit on all 16 datasets with a batch size of 32 and 20 epochs. A pre-trained BERT-Base model with a 128-unit feed-forward layer before the classification output layer was also fit with a batch size of 32 and 3 epochs. All models had a Word Embedding layer (word representations to capture the similarity between words) with an input length of 557 and an output dimension of 200. The BERT-Base model had both word embeddings and positional embeddings of dimension 512 by 768.

Since the focus of this study was to compare the performance of different models instead of optimizing a specific model, we tried to use the same and/or default hyperparameters for all models. There have been many studies and debates about the choice of hyperparameters [29–31]. The batch size can be any number between 1 and the number of samples in the training set. There are many factors that could affect the optimal choice of the batch size such as available computational resource, the size of the data, the choice of the optimizer and the learning rate. Generally speaking, the larger the batch size, the more likely the algorithm is to converge to the global minimum but more memory is required during the training process. When the batch size is too small, the model is more prone to noisiness and thus requires smaller learning rate for stability which results in more training steps and thus longer training time. There can be a sweet spot but it is dataset and model specific and requires a trial and error search. Commonly used batch sizes are 16, 32, and 64 for small and moderate-sized datasets. We chose to use 32 because our dataset was relatively small and the input sequences were long. Similar to the choice of the batch size, the choice of the number of epochs is also dataset and model specific. Larger number of epochs requires longer training time and could result in overfitting while small number of epochs could result in underfitting. We chose to use 20 epochs for all models except for BERT (3 epochs). The authors of BERT recommended 2–4 epochs for fine-tuning BERT [18]. Dropout was used to mitigate the overfitting problem. Commonly used dropout rates are between 0.1 and 0.5. We chose to use 0.3 for all models. For other hyperparameters such as learning rate, activation function, and optimizer, we chose to use the default values for all models.

Table 3 shows the architecture information of the seven models. The models were evaluated in terms of AUC-ROC (Area Under the Curve of the Receiver Operating Characteristic), AUC-PR (Area Under the Curve of Precision and Recall), F1 Score, and Balanced Accuracy (detailed reports on these metrics including Precision, Recall, and Specificity are shown in Table 4–9 in the Appendix).

**Table 3:** Model architectures

Model	Number of Filters/Units/Encoders	Embedding Dimension	Max Sequence Length	Dropout	Activation Function	Optimizer	Total Parameters
CNN	8	200	557	0.3	ReLU	Adam	5.51 M
RNN	8	200	557	0.3	ReLU	Adam	5.50 M
GRU	8	200	557	0.3	ReLU	Adam	5.50 M
LSTM	8	200	557	0.3	ReLU	Adam	5.50 M
Bi-LSTM	8	200	557	0.3	ReLU	Adam	5.51 M
Transformer Encoder	1 encoder (2 heads)	200	557	0.3	ReLU	Adam	5.94 M
BERT-Base	12 encoders (12 heads)	768	512	0.3 (fine-tune layer)	ReLU (fine-tune layer)	Adam (fine-tune layer)	110 M

Word embeddings have replaced the traditional Bag-of-Words (BoW) representations (e.g. TF-IDF, Count Vectorization) and have become essential in NLP tasks. They are the projection of tokenized word vectors onto a real-valued embedding matrix that are learned from the data during training or pre-trained on large datasets. One advantage of word embeddings over BoW is their significantly lower dimension that at most equals the maximum length of the input sequences (versus the maximum number of unique words in the dataset for BoW). Another advantage is their dense feature (less zeros for word embeddings compared to the sparse feature of BoW) [32]. Pre-trained word embeddings are widely used in NLP tasks with small datasets because they better capture the semantic and syntactic meaning of a word since they are trained on large datasets. Many different models for creating pre-trained word embeddings such as Word2Vec, GloVe, fastText, and BioWordVec among others have been developed. In this study, we implemented the GloVe embeddings (dimension 200) and the BioWordVec embeddings (dimension 200) on all models except for the Transformer encoder and BERT-Base and no pre-trained word embeddings for all models (word embeddings of dimension 200 learned from data during training). Pre-trained word embeddings were not used on the Transformer encoder and BERT-Base in this study because they are not a priority in the design of Transformers and BERT additionally requires special tokens for input sequences such as [CLS] and [SEP]. GloVe was trained on five corpora of varying sizes, including Wikipedia, Gigaword and web data from Common Crawl5 [33]. BioWordVec embeddings were trained on biomedical text and a biomedical controlled vocabulary called Medical Subject Headings (MeSH) to accommodate for the NLP needs in the biomedical domain [34].

## Results

**Fig. 1:**
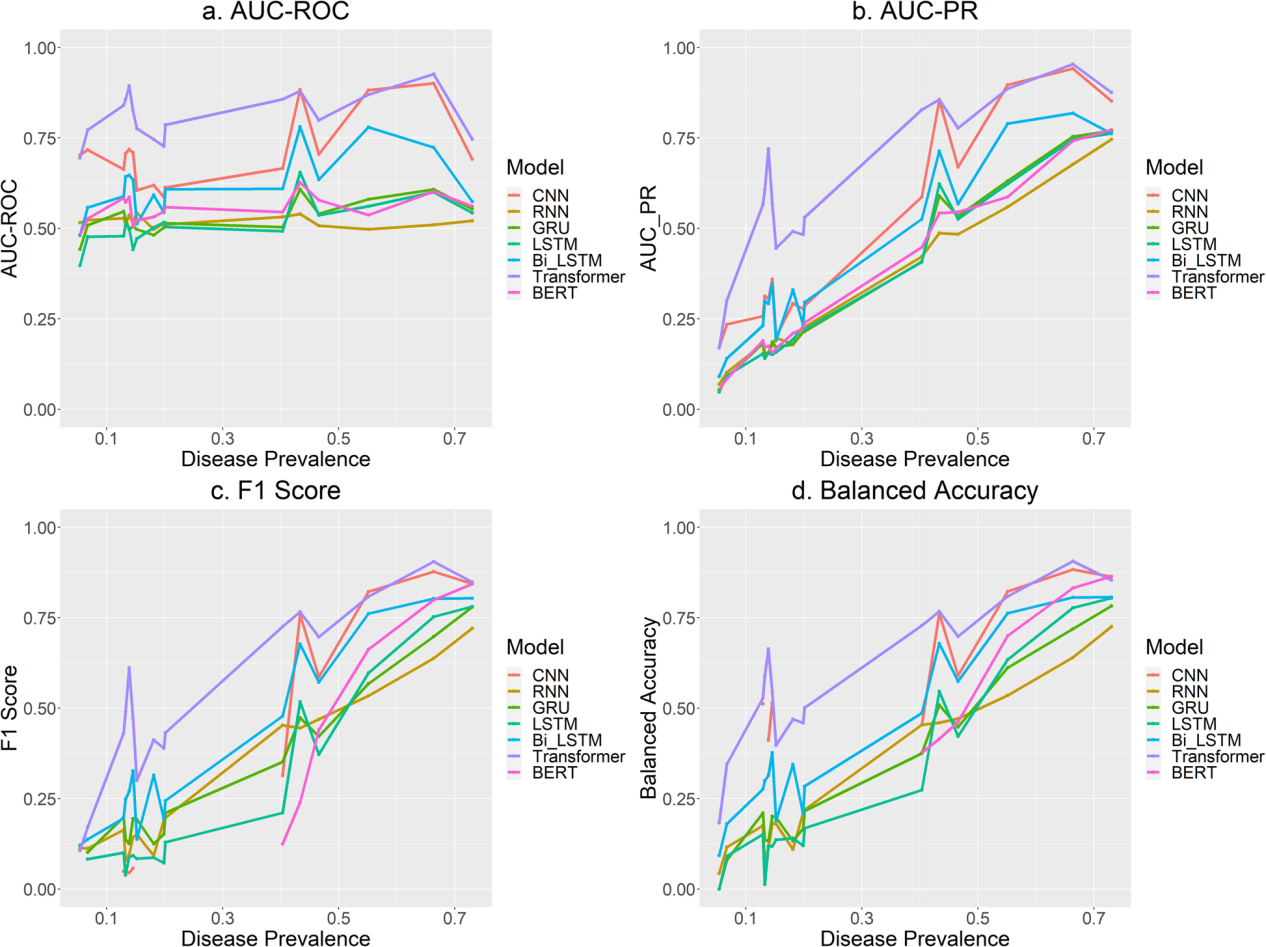
Model Performance. (**c**. F1 Score* and **d**. Balanced Accuracy*: some points in these graphs are missing due to NA values resulted from zero values for the True Positives in the highly imbalanced datasets)

Figure 1 (a, b, c, d) shows the AUC-ROC, AUC-PR, F1 Score, and Balanced Accuracy of the seven models for all 16 datasets ordered from lowest to highest according to the disease prevalence. The Transformer encoder produced the highest AUC-ROC for 13 datasets (the highest of the 13 is 0.926 for Diabetes), the highest AUC-PR for 14 datasets (the highest of the 14 is 0.954 for Diabetes), the highest F1 Score for 15 datasets (the highest of the 15 is 0.905 for Diabetes), and the highest Balanced Accuracy for 14 datasets (the highest of the 14 is 0.939 for Diabetes). CNN exceeded the Transformer encoder for 3 datasets in terms of AUC-ROC (0.702 for Hypertriglyceridemia, 0.883 for CHF, and 0.882 for CAD), 2 datasets in terms of AUC-PR (0.171 for Hypertriglyceridemia, and 0.897 for CAD), 1 dataset in terms of F1 Score (0.822 for CAD), and 2 datasets in terms of Balanced Accuracy (0.823 for CAD, and 0.863 for Hypertension). The values of the F1 Score and/or the Balanced Accuracy were unavailable for some models due to zero values for the True Positives (actual disease presence correctly predicted) which was encountered when the disease prevalence was less than 20%. The zero values for the True Positives occur when the algorithm predicts all positive cases as negative. Similarly, when a good portion of the positive cases (which can be a very small number in a small sample) are misclassified, the AUC-PR, F1 score, and the Balanced Accuracy would be low, while the AUC-ROC and the overall accuracy can still be high. This is not surprising because when the classes are highly imbalanced and the sample size is small, there are not many minority cases for the algorithms to learn the distinct characteristics of the minority class, and the cost for misclassifying minority cases is small even when all minority cases are misclassified.

Sordo et. al. reported that, as the sample size increases, machine learning algorithms (Naïve Bayes, SVM, and Decision Trees) show a substantial improvement in performance in predicting the smoking status of a patient from text excerpts extracted from narrative medical reports [35]. This is reasonable because the algorithms need to “learn” the latent information in the data by being exposed to a large enough amount of data in both classes. Given our small sample size (a total of 1,237 lengthy medical notes) and the fact that deep learning algorithms are data hungry, the performance of the Transformer encoder and the CNN algorithm are quite promising on the relatively balanced datasets. The datasets on which the Transformer encoder and the CNN algorithm performed poorly were all highly imbalanced and the other five models performed even worse on them. The low performance on the imbalanced datasets is a result of having too few samples in the minority class for the algorithms to learn from, or the number of minority cases in the training set not big enough to represent the minority class in the test set.

Table 4 shows how small the sample sizes are in the minority class in the training set, especially when the classes are highly imbalanced. For the datasets where the disease prevalence is less than 10%, the number of samples in the minority class in the training set is no more than 62 which is challenging for any algorithm to perform well. As shown in Fig. 1 (b, c, d), as the prevalence (or the number of samples in the minority class) increases, the performance of all models improved substantially in terms of AUC-PR, F1 score, and the Balanced Accuracy. Therefore, when used on big enough datasets (with a large enough number of samples in both classes), these algorithms could render excellent performance.

**Table 4:** Number of samples in each class in training and test sets

Disease	Prevalence	Training Set		Test Set	
		Disease Presence Presence	Disease Absence	Disease Presence	Disease Absence
Hypertriglyceridemia	5%	50	878	17	292
Venous Insufficiency	7%	62	865	21	289
Asthma	13%	123	805	41	268
Gout	13%	120	808	40	269
OSA	14%	129	799	43	266
PVD	15%	135	793	45	264
Gallstones	15%	141	787	47	262
OA	18%	168	760	56	253
GERD	20%	184	743	62	248
Depression	20%	187	741	62	247
Obesity	40%	374	554	125	184
CHF	43%	402	526	134	175
Hypercholesterolemia	47%	432	496	144	165
CAD	55%	512	416	170	139
Diabetes	66%	616	312	205	104
Hypertension	73%	677	250	226	84

**Fig. 2:**
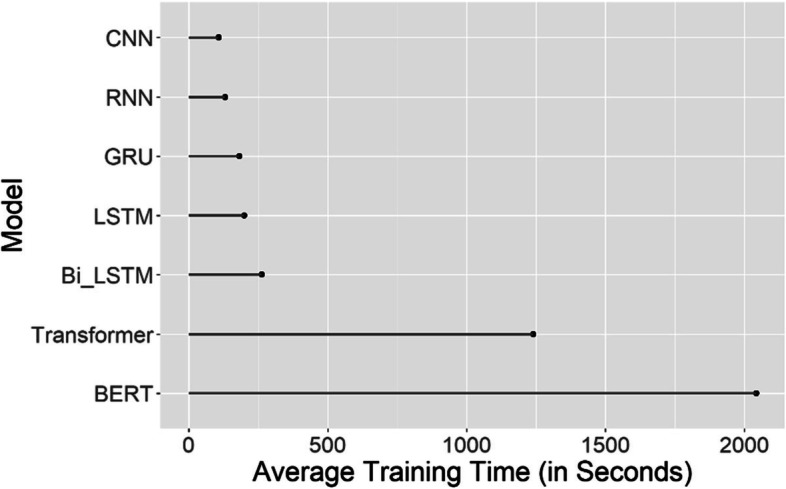
Average training time

Figure 2 displays the training time of all seven algorithms averaged over all 16 classification tasks (on a computer with Intel(R) Core(TM) i7-6560U CPU @ 2.20 GHz, and 16 GB RAM for). The CNN model ran consistently faster than all other models in all tasks. On average, CNN ran faster by as much as 17.6% than RNN which was the second fastest algorithm, 91.3% faster than the Transformer encoder and 94.7% faster than BERT-Base. The BERT-Base model is the most time consuming due to the large number of parameters being fed to the top layer even though it was only fine-tuned using 3 epochs.

**Fig. 3:**
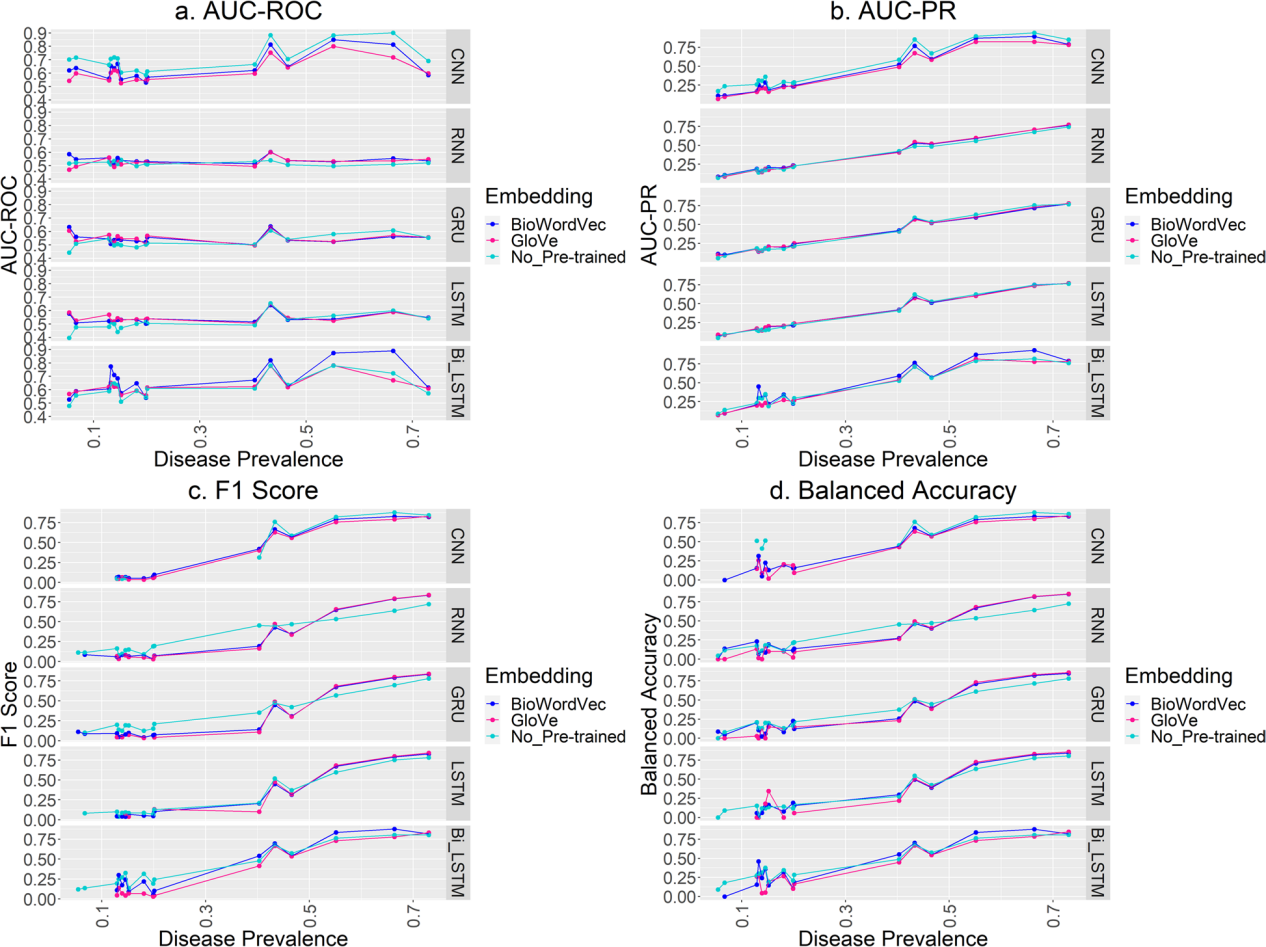
Model performance with and without Pre-trained Word Embeddings. (**c**. F1 Score* and **d**. Balanced Accuracy*: some points in these graphs are missing due to NaN values resulted from zero values for the True Positives in the highly imbalanced datasets)

Figure 3 shows the model performance with and without pre-trained word embeddings. There was slight improvement in terms of F1 Score, and Balanced Accuracy for RNN, GRU, and LSTM with both the GloVe and BioWordVec embeddings when the disease prevalence is greater than 50%. BioWordVec embeddings performed slightly better than GloVe embeddings in most cases, and the improvement is the most significant for the Bi-LSTM model. For the CNN model, the performance is better without pre-trained word embeddings.

**Fig. 4:**
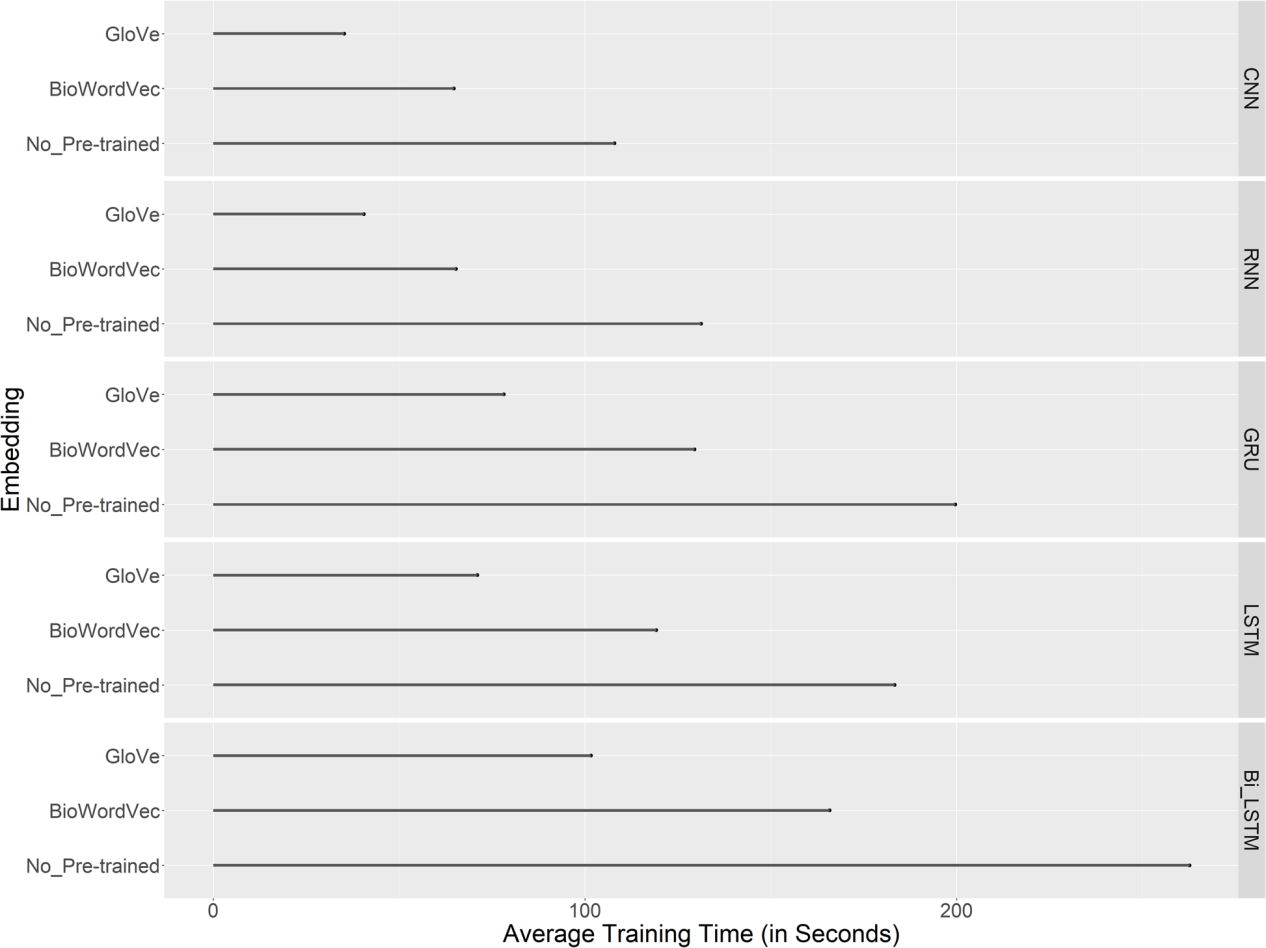
Average training time with and without pre-trained word embeddings

Figure 4 shows the training time of the models with and without pre-trained word embeddings averaged over all iterations and all datasets. Both pre-trained word embeddings shortened the training time significantly for all models. Models with the GloVe embeddings ran the fastest. The training time of Bi-LSTM was shortened by as much as 61% with the GloVe embeddings than without pre-trained embeddings.

## Discussion

Medical notes are often lengthy and thus constitute high-dimensional data. High-dimensional data (with a relatively small sample size) can be challenging due to its inevitable overfitting problems. The longest discharge summary in our data had 2,098 words after cleaning while the sample size was only 1,237. This issue was mitigated by the following treatments of the data. First, after tokenizing, the discharge notes of different lengths were forced into the same arbitrary length (557 in our study) by truncating the longer notes and padding the shorter notes with 0 s. There was no formula for the choice of the length, but Ying Wen et al. reported that a length that is close to the average length of the texts in the training set generally produces better results. If the length is too small, it will result in a great loss of information; if the length is too large, it will lead to sparse data in shorter notes and will include more noise from the longer notes [32]. Second, a relatively small number of epochs (20 epochs) was used to train the models to avoid the models memorizing the training data which is another source of overfitting with high-dimensional data.

In this study, the Transformer encoder stood out among all models in nearly all class imbalance scenarios. Its strength lies in the self-attention feature where all other words in the sequence are considered at once when encoding a specific word which effectively resolves the issue of “forgetting” information from previous words in long sequences that recurrent networks often encounter. CNN also outperformed the other five models and achieved a comparable performance to the Transformer encoder when the disease prevalence is close to or greater than 50%. Somewhat surprisingly, BERT-Base, as a powerful NLP model, performed poorly in all scenarios. It is likely due to the fact that it was trained on general text, not on medical text and thus failed to capture the information and the relationships of medical words. In addition, the maximum sequence length allowed in BERT-Base is 512 while the average length of the discharge summary notes in the dataset is 557 which could lead to too much loss of information.

The results for the five models with different word embeddings show that the BioWordVec embeddings slightly improved the performance of the Bi-LSTM model for some datasets. In general, models with BioWordVec embeddings performed slightly better than those with GloVe embeddings which is reasonable since the BioWordVec embeddings were trained on biomedical text while Glove embeddings were trained on corpora that are not in the biomedical domain. The reason that the improvement with BioWordVec was not quite noticeable may be due to the fact that many medical words (especially medication names such as zanflex, fondapurinox, diurhesis) and mis-spelled words (such as dopthromycin, anestheteic, amoxicil) in the dataset were still not recognized by BioWordVec. As expected, even more words were not recognized by GloVe which presumably contributed to the faster training time.

When the classes were highly imbalanced (disease prevalence lower than 30% or higher than 70%), all models performed poorly with very low AUC-PR and very low F1 Score and Balanced Accuracy (or even no available F1 Score and/or Balanced Accuracy). This is mainly due to unequal misclassification costs where misclassifying the minority class does not result in too much cost. This is especially true when the dataset is small and there are too few samples in the minority class to matter when misclassified. When the dataset is large, under-sampling the majority class, over-sampling the minority class, or a combination of the two can be used to balance the classes.

For small datasets, if collecting more data from the minority class is not feasible, using data augmentation (SMOTE, back-translation, random swap, and random deletion, etc.) to increase the number of samples in the minority class may help improve the performance of the models [19, 23–25]. In addition, for lengthy text data, the length of the text sequence to be fed into the model may also affect model performance since a short sequence may lose too much information and a long sequence may result in sparse data and introduce more noise as well as longer training time and overfitting problems [36–38]. If medical expert consultation is available, applying text preprocessing methods such as those implemented in some of the top 10 i2b2 challenge solutions should also help improve the model performance [24]. For example, abbreviations are very common in medical notes, and expanding them should help improve the results since otherwise they are often treated as unknown words and thus not contributing any information. In addition, most drug names are also not recognized and tagging drug names that are strongly indicative of a certain disease will also help improve prediction accuracy.

## Conclusion

In the binary text classification tasks studied, the Transformer encoder stood out among all algorithms studied (in terms of model performance metrics such as AUC-ROC, AUC-PR, F1 Score, and Balanced Accuracy). When the classes were more balanced, the CNN model performed equally well with markedly shorter training time. When the dataset was highly imbalanced with the positive class (disease presence) as the minority, AUC-ROC may be inflated, and AUC-PR may be a more reliable metric to evaluate model performance. In turn, when the dataset is highly imbalanced with the negative class (disease absence) as the minority, AUC-ROC may be a more accurate measure of model performance. In addition, domain specific pre-trained word embeddings such as BioBERT [39] and ClinicalBERT [40, 41] may help yield better results since the word embeddings are trained on medical text using the powerful BERT model. In summary, for classification tasks on medical notes, Transformer encoders are the best choice if the computation resource is not an issue. Otherwise, when the classes are relatively balanced, CNNs are a leading candidate because of its comparable performance and computational efficiency.

## Supplementary Information

**Additional file 1:** **Appendix: Table 4.** Model Performance Evaluation Metrics (Average of 10 Iterations). **Table 5.** Model Performance Evaluation Metrics (Average of 10 Iterations) - CNN with/without Pre-trained Word Embeddings. **Table 6.** Model Performance Evaluation Metrics (Average of 10 Iterations) - RNN with/without Pre-trained Word Embeddings. **Table 7.** Model Performance Evaluation Metrics (Average of 10 Iterations) - GRU with/without Pre-trained Word Embeddings. **Table 8.** Model Performance Evaluation Metrics (Average of 10 Iterations) - LSTM with/without Pre-trained Word Embeddings. **Table 9.** Model Performance Evaluation Metrics (Average of 10 Iterations) - Bi-LSTM with/without Pre-trained Word Embeddings.

## Data Availability

The data that support the findings in this study are derived from datasets publicly available after simple steps for approval on https://portal.dbmi.hms.harvard.edu/projects/n2c2-nlp/. The code for this study is available on https://github.com/Hanna520/medical_notes_AI.git.

## Abbreviations

AI: Artificial Intelligence
NLP: Natural Language Processing
CNN: Convolutional Neural Network
RNN: Recurrent Neural Network
GRU: Gated Recurrent Unit
LSTM: Long Short-Term Memory
Bi-LSTM: Bi-directional Long Short-Term Memory
BERT: Bi-directional Encoder Representations from Transformers
BoW: Bag-of-Words
AUC-ROC: Area Under the Curve of the Receiver Operating Characteristic
AUC-PR: Area Under the Curve of Precision and Recall
OSA: Obstructive Sleep Apnea
PVD: Peripheral Vascular Disease
OA: Osteo Arthritis
GERD: Gastroesophageal Reflux Disease
CHF: Congestive Heart Failure
CAD: Coronary Artery Disease
SMOTE: Synthetic Minority Oversampling Technique

## References

[1] Feder A, Vainstein D, Rosenfeld R, Hartman T, Hassidim A, Matias Y (2020). Active deep learning to detect demographic traits in free-form clinical notes. J Biomed Inform.

[2] Miotto R, Percha BL, Glicksberg BS, Lee HC, Cruz L, Dudley JT, Nabeel I (2020). Identifying acute low back pain episodes in primary care practice from clinical notes: Observational study. JMIR Med Inform.

[3] Gunjal H, Patel P, Thaker K, Nagrecha A, Mohammed S, Marchawala A. Text Summarization and classification of clinical discharge summaries using deep learning. 2020.

[4] Ye J, Yao L, Shen J, Janarthanam R, Luo Y (2020). Predicting mortality in critically ill patients with diabetes using machine learning and clinical notes. BMC Med Inform Decis Mak.

[5] Yang S, Yu X, Zhou Y. LSTM and GRU neural network performance comparison study: Taking Yelp review dataset as an example. In: 2020 International workshop on electronic communication and artificial intelligence (IWECAI). 2020. p. 98–101.

[6] Girgis S, Amer E, Gadallah M. Deep learning algorithms for detecting fake news in online text. In: 2018 13th International Conference on Computer Engineering and Systems (ICCES). 2018. p. 93–7.

[7] Onan A. Sentiment analysis on product reviews based on weighted word embeddings and deep neural networks. Concurrency and Computation: Practice and Experience. 2020;e5909.

[8] Kim H, Jeong YS (2019). Sentiment classification using convolutional neural networks. Appl Sci.

[9] Hughes M, Li I, Kotoulas S, Suzumura T. Medical text classification using convolutional neural networks. In: Informatics for Health: Connected Citizen-Led Wellness and Population Health. IOS Press; 2017. p. 246–50. 28423791

[10] Widiastuti NI. Convolution neural network for text mining and natural language processing. In: IOP Conference Series: Materials Science and Engineering. 2019. p. 52010.

[11] Banerjee I, Ling Y, Chen MC, Hasan SA, Langlotz CP, Moradzadeh N, Chapman B, Amrhein T, Mong D, Rubin DL (2019). Comparative effectiveness of convolutional neural network (CNN) and recurrent neural network (RNN) architectures for radiology text report classification. Artif Intell Med..

[12] Hijazi S, Kumar R, Rowen C (2015). Using convolutional neural networks for image recognition.

[13] Li Q, Cai W, Wang X, Zhou Y, Feng DD, Chen M. Medical image classification with convolutional neural network. In: 2014 13th international conference on control automation robotics & vision (ICARCV). 2014. p. 844–8.

[14] Liu Z, Huang H, Lu C, Lyu S. Multichannel cnn with attention for text classification. arXiv preprint arXiv:200616174. 2020;

[15] Zhao W, Joshi T, Nair VN, Sudjianto A. Shap values for explaining cnn-based text classification models. arXiv preprint arXiv:200811825. 2020;

[16] Cheng H, Yang X, Li Z, Xiao Y, Lin Y. Interpretable text classification using CNN and max-pooling. arXiv preprint arXiv:191011236. 2019;

[17] Vaswani A, Shazeer N, Parmar N, Uszkoreit J, Jones L, Gomez AN, Kaiser Ł, Polosukhin I. Attention is all you need. In: Advances in neural information processing systems. 2017. p. 5998–6008.

[18] Devlin J, Chang MW, Lee K, Toutanova K. Bert: Pre-training of deep bidirectional transformers for language understanding. arXiv preprint arXiv:181004805. 2018;

[19] Samghabadi NS, Patwa P, Srinivas P, Mukherjee P, Das A, Solorio T. Aggression and misogyny detection using BERT: A multi-task approach. In: Proceedings of the Second Workshop on Trolling, Aggression and Cyberbullying. 2020. p. 126–31.

[20] Gao Z, Feng A, Song X, Wu X (2019). Target-dependent sentiment classification with BERT. IEEE Access.

[21] Geng Z, Yan H, Qiu X, Huang X. fastHan: A BERT-based Multi-Task Toolkit for Chinese NLP. arXiv preprint arXiv:200908633. 2020;

[22] Zhang J, Chang W cheng, Yu H fu, Dhillon I. Fast multi-resolution transformer fine-tuning for extreme multi-label text classification. Advances in Neural Information Processing Systems. 2021;34.

[23] Harvard University i2b2 Obesity Challenge 2008 Data [Internet]. [cited 2022 Apr 28]. Available from: https://portal.dbmi.hms.harvard.edu/projects/n2c2-nlp/.

[24] Uzuner Ö (2009). Recognizing obesity and comorbidities in sparse data. J Am Med Inform Assoc.

[25] Ware H, Mullett CJ, Jagannathan V (2009). Natural language processing framework to assess clinical conditions. J Am Med Inform Assoc.

[26] Yang H, Spasic I, Keane JA, Nenadic G (2009). A text mining approach to the prediction of disease status from clinical discharge summaries. J Am Med Inform Assoc.

[27] Solt I, Tikk D, Gál V, Kardkovács ZT (2009). Semantic classification of diseases in discharge summaries using a context-aware rule-based classifier. J Am Med Inform Assoc.

[28] Schuster M, Nakajima K. Japanese and Korean voice search. In: 2012 IEEE International Conference on Acoustics, Speech and Signal Processing (ICASSP). 2012. p. 5149–52.

[29] Jastrzebski S, Kenton Z, Arpit D, Ballas N, Fischer A, Bengio Y, Storkey A. Three factors influencing minima in sgd. arXiv preprint arXiv:171104623. 2017;

[30] Kandel I, Castelli M (2020). The effect of batch size on the generalizability of the convolutional neural networks on a histopathology dataset. ICT express.

[31] Smith SL, Kindermans PJ, Ying C, Le Q v. Don’t decay the learning rate, increase the batch size. arXiv preprint arXiv:171100489. 2017;

[32] Almeida F, Xexéo G. Word embeddings: A survey. arXiv preprint arXiv:190109069. 2019;

[33] Pennington J, Socher R, Manning CD. Glove: Global vectors for word representation. In: Proceedings of the 2014 conference on empirical methods in natural language processing (EMNLP). 2014. p. 1532–43.

[34] Zhang Y, Chen Q, Yang Z, Lin H, Lu Z (2019). BioWordVec, improving biomedical word embeddings with subword information and MeSH. Sci Data.

[35] Sordo M, Zeng Q. On sample size and classification accuracy: A performance comparison. In: International Symposium on Biological and Medical Data Analysis. 2005. p. 193–201.

[36] Wen Y, Zhang W, Luo R, Wang J. Learning text representation using recurrent convolutional neural network with highway layers. arXiv preprint arXiv:160606905. 2016;

[37] Ibrahim M, Torki M, El-Makky N. Imbalanced toxic comments classification using data augmentation and deep learning. In: 2018 17th IEEE international conference on machine learning and applications (ICMLA). 2018. p. 875–8.

[38] Lauren P, Qu G, Watta P. Convolutional neural network for clinical narrative categorization. In: 2017 IEEE International Conference on Big Data (Big Data). 2017. p. 2001–8.

[39] Lee J, Yoon W, Kim S, Kim D, Kim S, So CH, Kang J (2020). BioBERT: a pre-trained biomedical language representation model for biomedical text mining. Bioinformatics.

[40] Alsentzer E, Murphy JR, Boag W, Weng WH, Jin D, Naumann T, McDermott M. Publicly available clinical BERT embeddings. arXiv preprint arXiv:190403323. 2019;

[41] Huang K, Altosaar J, Ranganath R. Clinicalbert: Modeling clinical notes and predicting hospital readmission. arXiv preprint arXiv:190405342. 2019;

