# Supplementary material for: A comparative study on deep learning models for text classification of unstructured medical notes with various levels of class imbalance
Source: BMC Med Res Methodol. 2022 Jul 2;22:181. doi: 10.1186/s12874-022-01665-y (PMC9250736; doi:10.1186/s12874-022-01665-y)
Supplement: Supplementary file 1 — Additional file 1: Appendix: Table 4. Model Performance Evaluation Metrics (Average of 10 Iterations). Table 5. Model Performance Evaluation Metrics (Average of 10 Iterations) - CNN with/without Pre-trained Word Embeddings. Table 6. Model Performance Evaluation Metrics (Average of 10 Iterations) - RNN with/without Pre-trained Word Embeddings. Table 7. Model Performance Evaluation Metrics (Average of 10 Iterations) - GRU with/without Pre-trained Word Embeddings. Table 8. Model Performance Evaluation Metrics (Average of 10 Iterations) - LSTM with/without Pre-trained Word Embeddings. Table 9. Model Performance Evaluation Metrics (Average of 10 Iterations) - Bi-LSTM with/without Pre-trained Word Embeddings. [file 12874_2022_1665_MOESM1_ESM.docx]

**Appendix**

**Table 4. Model Performance Evaluation Metrics (Average of 10 Iterations)**

| **Disease** | **Percent Disease** | **Model** | **AUC- ROC** | **AUC- PR** | **Balanced**  **Accuracy** | **Precision** | **Recall** | **Specificity** | **F1 Score** | **Running Time (Seconds)** |
| --- | --- | --- | --- | --- | --- | --- | --- | --- | --- | --- |
| Hypertrigly- ceridemia | 5% | CNN | 0.702 | 0.171 | NA | NA | 0.000 | 1.000 | NA | 112 |
|  |  | RNN | 0.516 | 0.070 | 0.044 | 0.041 | 0.041 | 0.976 | 0.113 | 142 |
|  |  | GRU | 0.442 | 0.054 | 0.000 | 0.000 | 0.000 | 0.983 | NA | 205 |
|  |  | LSTM | 0.397 | 0.048 | 0.000 | 0.000 | 0.000 | 0.998 | NA | 187 |
|  |  | Bi-LSTM | 0.481 | 0.091 | 0.093 | 0.144 | 0.041 | 0.986 | 0.121 | 266 |
|  |  | Transformer | 0.695 | 0.170 | 0.183 | 0.333 | 0.029 | 0.997 | 0.106 | 1205 |
|  |  | BERT | 0.482 | 0.054 | NA | NA | 0.000 | 1.000 | NA | 2053 |
| Venous Insufficiency | 7% | CNN | 0.717 | 0.235 | NA | NA | 0.000 | 1.000 | NA | 104 |
|  |  | RNN | 0.523 | 0.102 | 0.116 | 0.175 | 0.057 | 0.965 | 0.112 | 127 |
|  |  | GRU | 0.509 | 0.087 | 0.080 | 0.102 | 0.057 | 0.963 | 0.102 | 191 |
|  |  | LSTM | 0.477 | 0.094 | 0.091 | 0.161 | 0.019 | 0.992 | 0.083 | 175 |
|  |  | Bi-LSTM | 0.558 | 0.140 | 0.180 | 0.280 | 0.071 | 0.988 | 0.138 | 252 |
|  |  | Transformer | 0.772 | 0.302 | 0.345 | 0.600 | 0.081 | 0.996 | 0.171 | 1234 |
|  |  | BERT | 0.527 | 0.084 | NA | NA | 0.000 | 1.000 | NA | 2063 |
| Asthma | 13% | CNN | 0.663 | 0.257 | NA | 1.000 | 0.003 | 1.000 | 0.049 | 106 |
|  |  | RNN | 0.528 | 0.181 | 0.083 | 0.198 | 0.153 | 0.910 | 0.164 | 136 |
|  |  | GRU | 0.547 | 0.183 | 0.136 | 0.240 | 0.180 | 0.916 | 0.199 | 197 |
|  |  | LSTM | 0.479 | 0.154 | 0.013 | 0.245 | 0.058 | 0.971 | 0.100 | 180 |
|  |  | Bi-LSTM | 0.589 | 0.232 | 0.300 | 0.423 | 0.130 | 0.974 | 0.196 | 276 |
|  |  | Transformer | 0.840 | 0.566 | 0.588 | 0.742 | 0.313 | 0.985 | 0.431 | 1226 |
|  |  | BERT | 0.572 | 0.172 | NA | NA | 0.000 | 1.000 | NA | 1949 |
| Gout | 13% | CNN | 0.707 | 0.313 | 0.513 | NA | 0.000 | 1.000 | NA | 104 |
|  |  | RNN | 0.511 | 0.141 | 0.175 | 0.129 | 0.037 | 0.962 | 0.080 | 126 |
|  |  | GRU | 0.527 | 0.156 | 0.210 | 0.174 | 0.098 | 0.932 | 0.137 | 192 |
|  |  | LSTM | 0.513 | 0.140 | 0.151 | 0.021 | 0.005 | 0.980 | 0.039 | 178 |
|  |  | Bi-LSTM | 0.643 | 0.300 | 0.276 | 0.417 | 0.183 | 0.960 | 0.248 | 255 |
|  |  | Transformer | 0.856 | 0.606 | 0.527 | 0.816 | 0.361 | 0.987 | 0.491 | 1226 |
|  |  | BERT | 0.584 | 0.189 | NA | NA | 0.000 | 1.000 | NA | 2319 |
| OSA | 14% | CNN | 0.719 | 0.306 | 0.412 | 0.800 | 0.012 | 0.999 | 0.045 | 105 |
|  |  | RNN | 0.536 | 0.156 | 0.112 | 0.152 | 0.072 | 0.937 | 0.096 | 129 |
|  |  | GRU | 0.497 | 0.155 | 0.133 | 0.158 | 0.107 | 0.911 | 0.125 | 193 |
|  |  | LSTM | 0.500 | 0.155 | 0.120 | 0.186 | 0.053 | 0.961 | 0.089 | 181 |
|  |  | Bi-LSTM | 0.647 | 0.292 | 0.314 | 0.412 | 0.216 | 0.943 | 0.270 | 251 |
|  |  | Transformer | 0.894 | 0.720 | 0.663 | 0.846 | 0.481 | 0.985 | 0.611 | 1230 |
|  |  | BERT | 0.587 | 0.175 | NA | NA | 0.000 | 1.000 | NA | 1771 |

| PVD | 15% | CNN | 0.710 | 0.360 | NA | 1.000 | 0.009 | 1.000 | 0.057 | 104 |
| --- | --- | --- | --- | --- | --- | --- | --- | --- | --- | --- |
|  |  | RNN | 0.514 | 0.175 | 0.180 | 0.217 | 0.147 | 0.872 | 0.144 | 125 |
|  |  | GRU | 0.506 | 0.186 | 0.198 | 0.231 | 0.171 | 0.903 | 0.195 | 197 |
|  |  | LSTM | 0.442 | 0.151 | 0.136 | 0.190 | 0.047 | 0.972 | 0.092 | 183 |
|  |  | Bi-LSTM | 0.635 | 0.349 | 0.189 | 0.511 | 0.242 | 0.960 | 0.327 | 251 |
|  |  | Transformer | 0.825 | 0.569 | 0.398 | 0.741 | 0.349 | 0.978 | 0.471 | 1263 |
|  |  | BERT | 0.505 | 0.155 | NA | NA | 0.000 | 1.000 | NA | 2262 |
| Gallstones | 15% | CNN | 0.606 | 0.201 | 0.515 | NA | 0.000 | 1.000 | NA | 118 |
|  |  | RNN | 0.543 | 0.197 | 0.182 | 0.225 | 0.136 | 0.914 | 0.151 | 140 |
|  |  | GRU | 0.498 | 0.172 | 0.201 | 0.219 | 0.177 | 0.887 | 0.193 | 219 |
|  |  | LSTM | 0.472 | 0.158 | 0.118 | 0.219 | 0.053 | 0.965 | 0.084 | 199 |
|  |  | Bi-LSTM | 0.512 | 0.190 | 0.377 | 0.280 | 0.098 | 0.950 | 0.138 | 289 |
|  |  | Transformer | 0.776 | 0.445 | 0.545 | 0.591 | 0.204 | 0.975 | 0.300 | 1271 |
|  |  | BERT | 0.523 | 0.169 | NA | NA | 0.000 | 1.000 | NA | 1602 |
| OA | 18% | CNN | 0.619 | 0.293 | NA | NA | 0.000 | 1.000 | NA | 121 |
|  |  | RNN | 0.497 | 0.181 | 0.110 | 0.148 | 0.071 | 0.920 | 0.092 | 148 |
|  |  | GRU | 0.482 | 0.179 | 0.133 | 0.162 | 0.104 | 0.883 | 0.125 | 231 |
|  |  | LSTM | 0.502 | 0.193 | 0.141 | 0.191 | 0.091 | 0.911 | 0.087 | 212 |
|  |  | Bi-LSTM | 0.593 | 0.330 | 0.345 | 0.432 | 0.257 | 0.917 | 0.315 | 290 |
|  |  | Transformer | 0.746 | 0.492 | 0.469 | 0.632 | 0.307 | 0.961 | 0.412 | 1256 |
|  |  | BERT | 0.532 | 0.210 | NA | NA | 0.000 | 1.000 | NA | 2272 |
| GERD | 20% | CNN | 0.587 | 0.278 | NA | NA | 0.000 | 1.000 | NA | 108 |
|  |  | RNN | 0.515 | 0.215 | 0.220 | 0.197 | 0.227 | 0.763 | 0.191 | 134 |
|  |  | GRU | 0.502 | 0.214 | 0.216 | 0.212 | 0.121 | 0.883 | 0.152 | 208 |
|  |  | LSTM | 0.517 | 0.229 | 0.168 | 0.206 | 0.034 | 0.973 | 0.072 | 185 |
|  |  | Bi-LSTM | 0.544 | 0.233 | 0.284 | 0.257 | 0.158 | 0.887 | 0.193 | 264 |
|  |  | Transformer | 0.727 | 0.483 | 0.501 | 0.637 | 0.282 | 0.959 | 0.389 | 1243 |
|  |  | BERT | 0.547 | 0.224 | NA | NA | 0.000 | 1.000 | NA | 2135 |
| Depression | 20% | CNN | 0.613 | 0.286 | NA | NA | 0.000 | 1.000 | NA | 108 |
|  |  | RNN | 0.511 | 0.228 | 0.212 | 0.237 | 0.202 | 0.812 | 0.197 | 130 |
|  |  | GRU | 0.515 | 0.215 | 0.166 | 0.240 | 0.192 | 0.845 | 0.211 | 199 |
|  |  | LSTM | 0.504 | 0.221 | 0.120 | 0.245 | 0.092 | 0.933 | 0.129 | 184 |
|  |  | Bi-LSTM | 0.608 | 0.296 | 0.207 | 0.379 | 0.190 | 0.912 | 0.244 | 260 |
|  |  | Transformer | 0.786 | 0.531 | 0.459 | 0.681 | 0.321 | 0.961 | 0.432 | 1240 |
|  |  | BERT | 0.559 | 0.240 | NA | NA | 0.000 | 1.000 | NA | 2251 |
| Obesity | 40% | CNN | 0.666 | 0.587 | 0.453 | 0.700 | 0.206 | 0.944 | 0.314 | 107 |
|  |  | RNN | 0.532 | 0.422 | 0.454 | 0.435 | 0.474 | 0.582 | 0.453 | 128 |
|  |  | GRU | 0.504 | 0.409 | 0.375 | 0.408 | 0.342 | 0.658 | 0.351 | 195 |
|  |  | LSTM | 0.492 | 0.407 | 0.274 | 0.402 | 0.146 | 0.854 | 0.211 | 181 |
|  |  | Bi-LSTM | 0.610 | 0.526 | 0.486 | 0.537 | 0.435 | 0.744 | 0.478 | 258 |
|  |  | Transformer | 0.857 | 0.828 | 0.729 | 0.772 | 0.686 | 0.863 | 0.725 | 1232 |
|  |  | BERT | 0.545 | 0.448 | 0.377 | 0.679 | 0.022 | 0.986 | 0.124 | 2023 |

| CHF | 43% | CNN | 0.883 | 0.855 | 0.762 | 0.804 | 0.720 | 0.866 | 0.759 | 106 |
| --- | --- | --- | --- | --- | --- | --- | --- | --- | --- | --- |
|  |  | RNN | 0.540 | 0.487 | 0.460 | 0.491 | 0.428 | 0.631 | 0.445 | 126 |
|  |  | GRU | 0.608 | 0.590 | 0.509 | 0.622 | 0.396 | 0.807 | 0.473 | 194 |
|  |  | LSTM | 0.655 | 0.623 | 0.547 | 0.638 | 0.455 | 0.792 | 0.518 | 179 |
|  |  | Bi-LSTM | 0.781 | 0.714 | 0.679 | 0.690 | 0.668 | 0.767 | 0.677 | 258 |
|  |  | Transformer | 0.880 | 0.857 | 0.767 | 0.788 | 0.746 | 0.847 | 0.766 | 1237 |
|  |  | BERT | 0.627 | 0.542 | 0.415 | 0.637 | 0.173 | 0.896 | 0.240 | 2188 |
| Hyper- cholesterolemia | 47% | CNN | 0.705 | 0.670 | 0.590 | 0.635 | 0.546 | 0.722 | 0.586 | 107 |
|  |  | RNN | 0.507 | 0.484 | 0.471 | 0.466 | 0.476 | 0.527 | 0.470 | 134 |
|  |  | GRU | 0.540 | 0.534 | 0.448 | 0.528 | 0.368 | 0.705 | 0.422 | 196 |
|  |  | LSTM | 0.536 | 0.526 | 0.422 | 0.534 | 0.311 | 0.756 | 0.372 | 179 |
|  |  | Bi-LSTM | 0.635 | 0.568 | 0.574 | 0.562 | 0.587 | 0.601 | 0.572 | 270 |
|  |  | Transformer | 0.799 | 0.778 | 0.698 | 0.710 | 0.687 | 0.755 | 0.697 | 1241 |
|  |  | BERT | 0.578 | 0.545 | 0.462 | 0.498 | 0.427 | 0.640 | 0.440 | 2161 |
| CAD | 55% | CNN | 0.882 | 0.897 | 0.823 | 0.811 | 0.835 | 0.760 | 0.822 | 105 |
|  |  | RNN | 0.498 | 0.559 | 0.535 | 0.546 | 0.525 | 0.463 | 0.534 | 125 |
|  |  | GRU | 0.580 | 0.631 | 0.612 | 0.608 | 0.616 | 0.458 | 0.568 | 192 |
|  |  | LSTM | 0.561 | 0.623 | 0.634 | 0.590 | 0.678 | 0.392 | 0.597 | 176 |
|  |  | Bi-LSTM | 0.780 | 0.790 | 0.763 | 0.739 | 0.786 | 0.658 | 0.762 | 254 |
|  |  | Transformer | 0.870 | 0.887 | 0.809 | 0.803 | 0.815 | 0.753 | 0.809 | 1229 |
|  |  | BERT | 0.537 | 0.587 | 0.700 | 0.558 | 0.843 | 0.176 | 0.663 | 1976 |
| Diabetes | 66% | CNN | 0.901 | 0.942 | 0.883 | 0.815 | 0.952 | 0.568 | 0.878 | 106 |
|  |  | RNN | 0.510 | 0.678 | 0.640 | 0.665 | 0.616 | 0.387 | 0.638 | 126 |
|  |  | GRU | 0.608 | 0.754 | 0.719 | 0.716 | 0.721 | 0.394 | 0.698 | 194 |
|  |  | LSTM | 0.600 | 0.746 | 0.777 | 0.678 | 0.877 | 0.154 | 0.752 | 178 |
|  |  | Bi-LSTM | 0.724 | 0.819 | 0.806 | 0.760 | 0.853 | 0.461 | 0.803 | 253 |
|  |  | Transformer | 0.926 | 0.954 | 0.907 | 0.874 | 0.939 | 0.732 | 0.905 | 1272 |
|  |  | BERT | 0.602 | 0.742 | 0.832 | 0.666 | 0.999 | 0.006 | 0.799 | 1593 |
| Hypertension | 73% | CNN | 0.692 | 0.852 | 0.863 | 0.733 | 0.994 | 0.026 | 0.844 | 106 |
|  |  | RNN | 0.521 | 0.746 | 0.726 | 0.734 | 0.717 | 0.304 | 0.721 | 124 |
|  |  | GRU | 0.553 | 0.770 | 0.782 | 0.745 | 0.820 | 0.244 | 0.780 | 191 |
|  |  | LSTM | 0.543 | 0.763 | 0.804 | 0.736 | 0.873 | 0.148 | 0.781 | 176 |
|  |  | Bi-LSTM | 0.574 | 0.762 | 0.807 | 0.758 | 0.856 | 0.267 | 0.804 | 256 |
|  |  | Transformer | 0.746 | 0.876 | 0.854 | 0.786 | 0.922 | 0.324 | 0.848 | 1248 |
|  |  | BERT | 0.561 | 0.773 | 0.865 | 0.729 | 1.000 | 0.000 | 0.843 | 2091 |

**Table 5. Model Performance Evaluation Metrics (Average of 10 Iterations) - CNN with/without Pre-trained Word Embeddings**

| **Disease** | **Percent Disease** | **Embedding** | **AUC- ROC** | **AUC- PR** | **Balanced**  **Accuracy** | **Precision** | **Recall** | **Specificity** | **F1 Score** | **Running Time (Seconds)** |
| --- | --- | --- | --- | --- | --- | --- | --- | --- | --- | --- |
| Hypertrigly- ceridemia | 5% | No Pre-trained | 0.702 | 0.171 | NA | NA | 0.000 | 1.000 | NA | 112 |
|  |  | GloVe | 0.543 | 0.068 | NA | NA | 0.000 | 1.000 | NA | 34 |
|  |  | BioWordVec | 0.621 | 0.108 | NA | NA | 0.000 | 1.000 | NA | 50 |
| Venous Insufficiency | 7% | No Pre-trained | 0.717 | 0.235 | NA | NA | 0.000 | 1.000 | NA | 104 |
|  |  | GloVe | 0.600 | 0.096 | NA | NA | 0.000 | 1.000 | NA | 35 |
|  |  | BioWordVec | 0.638 | 0.112 | 0.000 | 0.000 | 0.000 | 1.000 | NA | 48 |
| Asthma | 13% | No Pre-trained | 0.663 | 0.257 | 0.513 | 1.000 | 0.003 | 1.000 | 0.049 | 106 |
|  |  | GloVe | 0.547 | 0.159 | 0.147 | 0.278 | 0.005 | 0.999 | 0.047 | 32 |
|  |  | BioWordVec | 0.559 | 0.165 | 0.156 | 0.292 | 0.010 | 0.995 | 0.061 | 63 |
| Gout | 13% | No Pre-trained | 0.707 | 0.313 | NA | NA | 0.000 | 1.000 | NA | 104 |
|  |  | GloVe | 0.604 | 0.195 | 0.258 | 0.500 | 0.005 | 0.999 | 0.047 | 32 |
|  |  | BioWordVec | 0.654 | 0.252 | 0.315 | 0.601 | 0.027 | 0.997 | 0.070 | 54 |
| OSA | 14% | No Pre-trained | 0.719 | 0.306 | 0.412 | 0.800 | 0.012 | 0.999 | 0.045 | 105 |
|  |  | GloVe | 0.623 | 0.210 | 0.088 | 0.167 | 0.007 | 0.997 | 0.066 | 34 |
|  |  | BioWordVec | 0.636 | 0.216 | 0.050 | 0.089 | 0.007 | 0.993 | 0.059 | 63 |
| PVD | 15% | No Pre-trained | 0.710 | 0.360 | 0.515 | 1.000 | 0.009 | 1.000 | 0.057 | 104 |
|  |  | GloVe | 0.615 | 0.206 | 0.140 | 0.267 | 0.009 | 0.992 | 0.056 | 40 |
|  |  | BioWordVec | 0.670 | 0.285 | 0.226 | 0.424 | 0.022 | 0.994 | 0.067 | 67 |
| Gallstones | 15% | No Pre-trained | 0.606 | 0.201 | NA | NA | 0.000 | 1.000 | NA | 118 |
|  |  | GloVe | 0.525 | 0.161 | 0.019 | 0.033 | 0.002 | 0.996 | 0.038 | 41 |
|  |  | BioWordVec | 0.553 | 0.184 | 0.130 | 0.250 | 0.009 | 0.996 | 0.054 | 72 |
| OA | 18% | No Pre-trained | 0.619 | 0.293 | NA | NA | 0.000 | 1.000 | NA | 121 |
|  |  | GloVe | 0.551 | 0.225 | 0.207 | 0.403 | 0.011 | 0.993 | 0.034 | 41 |
|  |  | BioWordVec | 0.581 | 0.238 | 0.198 | 0.374 | 0.020 | 0.991 | 0.052 | 52 |
| GERD | 20% | No Pre-trained | 0.587 | 0.278 | NA | NA | 0.000 | 1.000 | NA | 108 |
|  |  | GloVe | 0.546 | 0.245 | 0.191 | 0.354 | 0.029 | 0.985 | 0.057 | 38 |
|  |  | BioWordVec | 0.531 | 0.235 | 0.155 | 0.273 | 0.037 | 0.970 | 0.075 | 63 |
| Depression | 20% | No Pre-trained | 0.613 | 0.286 | NA | NA | 0.000 | 1.000 | NA | 108 |
|  |  | GloVe | 0.553 | 0.232 | 0.094 | 0.165 | 0.024 | 0.977 | 0.067 | 37 |
|  |  | BioWordVec | 0.570 | 0.245 | 0.158 | 0.269 | 0.047 | 0.973 | 0.095 | 67 |
| Obesity | 40% | No Pre-trained | 0.666 | 0.587 | 0.453 | 0.700 | 0.206 | 0.944 | 0.314 | 107 |
|  |  | GloVe | 0.598 | 0.489 | 0.430 | 0.520 | 0.341 | 0.783 | 0.401 | 33 |
|  |  | BioWordVec | 0.620 | 0.521 | 0.442 | 0.515 | 0.369 | 0.758 | 0.421 | 69 |
| CHF | 43% | No Pre-trained | 0.883 | 0.855 | 0.762 | 0.804 | 0.720 | 0.866 | 0.759 | 106 |
|  |  | GloVe | 0.752 | 0.673 | 0.634 | 0.651 | 0.618 | 0.745 | 0.629 | 34 |
|  |  | BioWordVec | 0.813 | 0.769 | 0.680 | 0.748 | 0.611 | 0.836 | 0.666 | 69 |
| Hyper- cholesterolemia | 47% | No Pre-trained | 0.705 | 0.670 | 0.590 | 0.635 | 0.546 | 0.722 | 0.586 | 107 |
|  |  | GloVe | 0.643 | 0.586 | 0.572 | 0.577 | 0.567 | 0.624 | 0.558 | 34 |
|  |  | BioWordVec | 0.649 | 0.594 | 0.574 | 0.576 | 0.572 | 0.631 | 0.571 | 67 |
| CAD | 55% | No Pre-trained | 0.882 | 0.897 | 0.823 | 0.811 | 0.835 | 0.760 | 0.822 | 105 |
|  |  | GloVe | 0.801 | 0.824 | 0.758 | 0.734 | 0.782 | 0.650 | 0.756 | 34 |
|  |  | BioWordVec | 0.851 | 0.872 | 0.792 | 0.800 | 0.785 | 0.755 | 0.791 | 72 |
| Diabetes | 66% | No Pre-trained | 0.901 | 0.942 | 0.883 | 0.815 | 0.952 | 0.568 | 0.878 | 106 |
|  |  | GloVe | 0.718 | 0.824 | 0.800 | 0.735 | 0.864 | 0.378 | 0.793 | 34 |
|  |  | BioWordVec | 0.814 | 0.894 | 0.829 | 0.795 | 0.864 | 0.552 | 0.827 | 79 |
| Hypertension | 73% | No Pre-trained | 0.692 | 0.852 | 0.863 | 0.733 | 0.994 | 0.026 | 0.844 | 106 |
|  |  | GloVe | 0.598 | 0.785 | 0.842 | 0.738 | 0.947 | 0.093 | 0.829 | 33 |
|  |  | BioWordVec | 0.586 | 0.789 | 0.832 | 0.738 | 0.926 | 0.115 | 0.821 | 81 |

**Table 6. Model Performance Evaluation Metrics (Average of 10 Iterations) - RNN with/without Pre-trained Word Embeddings**

| **Disease** | **Percent Disease** | **Embedding** | **AUC- ROC** | **AUC- PR** | **Balanced**  **Accuracy** | **Precision** | **Recall** | **Specificity** | **F1 Score** | **Running Time (Seconds)** |
| --- | --- | --- | --- | --- | --- | --- | --- | --- | --- | --- |
| Hypertrigly- ceridemia | 5% | No Pre-trained | 0.516 | 0.070 | 0.044 | 0.041 | 0.041 | 0.976 | 0.113 | 142 |
|  |  | GloVe | 0.471 | 0.076 | 0.000 | 0.000 | 0.000 | 0.999 | NA | 49 |
|  |  | BioWordVec | 0.586 | 0.084 | 0.000 | 0.000 | 0.000 | 0.996 | NA | 68 |
| Venous Insufficiency | 7% | No Pre-trained | 0.523 | 0.102 | 0.116 | 0.175 | 0.057 | 0.965 | 0.112 | 127 |
|  |  | GloVe | 0.494 | 0.088 | 0.000 | 0.000 | 0.000 | 0.999 | NA | 49 |
|  |  | BioWordVec | 0.549 | 0.107 | 0.137 | 0.250 | 0.010 | 0.999 | 0.087 | 65 |
| Asthma | 13% | No Pre-trained | 0.528 | 0.181 | 0.175 | 0.198 | 0.153 | 0.910 | 0.164 | 136 |
|  |  | GloVe | 0.561 | 0.174 | 0.133 | 0.235 | 0.028 | 0.979 | 0.067 | 36 |
|  |  | BioWordVec | 0.560 | 0.188 | 0.233 | 0.444 | 0.013 | 0.994 | 0.059 | 65 |
| Gout | 13% | No Pre-trained | 0.511 | 0.141 | 0.083 | 0.129 | 0.037 | 0.962 | 0.080 | 126 |
|  |  | GloVe | 0.515 | 0.141 | 0.013 | 0.018 | 0.002 | 0.991 | 0.033 | 40 |
|  |  | BioWordVec | 0.521 | 0.147 | 0.069 | 0.125 | 0.002 | 0.998 | 0.044 | 66 |
| OSA | 14% | No Pre-trained | 0.536 | 0.156 | 0.112 | 0.152 | 0.072 | 0.937 | 0.096 | 129 |
|  |  | GloVe | 0.492 | 0.145 | 0.000 | 0.000 | 0.000 | 0.996 | NA | 42 |
|  |  | BioWordVec | 0.512 | 0.162 | 0.109 | 0.166 | 0.047 | 0.958 | 0.084 | 65 |
| PVD | 15% | No Pre-trained | 0.514 | 0.175 | 0.182 | 0.217 | 0.147 | 0.872 | 0.144 | 125 |
|  |  | GloVe | 0.530 | 0.189 | 0.170 | 0.306 | 0.031 | 0.988 | 0.078 | 49 |
|  |  | BioWordVec | 0.558 | 0.186 | 0.086 | 0.143 | 0.027 | 0.980 | 0.081 | 64 |
| Gallstones | 15% | No Pre-trained | 0.543 | 0.197 | 0.180 | 0.225 | 0.136 | 0.914 | 0.151 | 140 |
|  |  | GloVe | 0.510 | 0.178 | 0.102 | 0.188 | 0.013 | 0.990 | 0.058 | 49 |
|  |  | BioWordVec | 0.540 | 0.207 | 0.192 | 0.353 | 0.026 | 0.992 | 0.064 | 65 |
| OA | 18% | No Pre-trained | 0.497 | 0.181 | 0.110 | 0.148 | 0.071 | 0.920 | 0.092 | 148 |
|  |  | GloVe | 0.527 | 0.194 | 0.099 | 0.188 | 0.009 | 0.989 | 0.052 | 51 |
|  |  | BioWordVec | 0.533 | 0.204 | 0.114 | 0.196 | 0.029 | 0.974 | 0.084 | 69 |
| GERD | 20% | No Pre-trained | 0.515 | 0.215 | 0.212 | 0.197 | 0.227 | 0.763 | 0.191 | 134 |
|  |  | GloVe | 0.527 | 0.227 | 0.022 | 0.042 | 0.002 | 0.995 | 0.030 | 40 |
|  |  | BioWordVec | 0.530 | 0.233 | 0.114 | 0.222 | 0.005 | 0.995 | 0.031 | 65 |
| Depression | 20% | No Pre-trained | 0.511 | 0.228 | 0.220 | 0.237 | 0.202 | 0.812 | 0.197 | 130 |
|  |  | GloVe | 0.524 | 0.229 | 0.096 | 0.172 | 0.019 | 0.985 | 0.069 | 38 |
|  |  | BioWordVec | 0.531 | 0.228 | 0.135 | 0.231 | 0.035 | 0.975 | 0.074 | 65 |
| Obesity | 40% | No Pre-trained | 0.532 | 0.422 | 0.454 | 0.435 | 0.474 | 0.582 | 0.453 | 128 |
|  |  | GloVe | 0.497 | 0.407 | 0.264 | 0.424 | 0.104 | 0.901 | 0.165 | 34 |
|  |  | BioWordVec | 0.515 | 0.417 | 0.274 | 0.415 | 0.133 | 0.870 | 0.194 | 65 |
| CHF | 43% | No Pre-trained | 0.540 | 0.487 | 0.460 | 0.491 | 0.428 | 0.631 | 0.445 | 126 |
|  |  | GloVe | 0.601 | 0.544 | 0.493 | 0.580 | 0.405 | 0.767 | 0.470 | 35 |
|  |  | BioWordVec | 0.603 | 0.529 | 0.465 | 0.588 | 0.341 | 0.816 | 0.429 | 65 |
| Hyper- cholesterolemia | 47% | No Pre-trained | 0.507 | 0.484 | 0.471 | 0.466 | 0.476 | 0.527 | 0.470 | 134 |
|  |  | GloVe | 0.541 | 0.523 | 0.408 | 0.569 | 0.247 | 0.831 | 0.337 | 34 |
|  |  | BioWordVec | 0.539 | 0.515 | 0.401 | 0.551 | 0.251 | 0.823 | 0.342 | 65 |
| CAD | 55% | No Pre-trained | 0.498 | 0.559 | 0.535 | 0.546 | 0.525 | 0.463 | 0.534 | 125 |
|  |  | GloVe | 0.533 | 0.599 | 0.681 | 0.555 | 0.806 | 0.202 | 0.655 | 34 |
|  |  | BioWordVec | 0.529 | 0.593 | 0.669 | 0.551 | 0.787 | 0.207 | 0.647 | 65 |
| Diabetes | 66% | No Pre-trained | 0.510 | 0.678 | 0.640 | 0.665 | 0.616 | 0.387 | 0.638 | 126 |
|  |  | GloVe | 0.538 | 0.709 | 0.821 | 0.662 | 0.981 | 0.009 | 0.790 | 34 |
|  |  | BioWordVec | 0.554 | 0.709 | 0.816 | 0.664 | 0.968 | 0.028 | 0.787 | 63 |
| Hypertension | 73% | No Pre-trained | 0.521 | 0.746 | 0.726 | 0.734 | 0.717 | 0.304 | 0.721 | 124 |
|  |  | GloVe | 0.549 | 0.773 | 0.854 | 0.734 | 0.973 | 0.049 | 0.837 | 34 |
|  |  | BioWordVec | 0.538 | 0.765 | 0.850 | 0.732 | 0.968 | 0.046 | 0.833 | 63 |

**Table 7. Model Performance Evaluation Metrics (Average of 10 Iterations) - GRU with/without Pre-trained Word Embeddings**

| **Disease** | **Percent Disease** | **Embedding** | **AUC- ROC** | **AUC- PR** | **Balanced**  **Accuracy** | **Precision** | **Recall** | **Specificity** | **F1 Score** | **Running Time (Seconds)** |
| --- | --- | --- | --- | --- | --- | --- | --- | --- | --- | --- |
| Hypertrigly- ceridemia | 5% | No Pre-trained | 0.442 | 0.054 | 0.000 | 0.000 | 0.000 | 0.983 | NA | 205 |
|  |  | GloVe | 0.606 | 0.097 | 0.000 | 0.000 | 0.000 | 1.000 | NA | 93 |
|  |  | BioWordVec | 0.632 | 0.110 | 0.088 | 0.167 | 0.006 | 0.998 | 0.111 | 128 |
| Venous Insufficiency | 7% | No Pre-trained | 0.509 | 0.087 | 0.080 | 0.102 | 0.057 | 0.963 | 0.102 | 191 |
|  |  | GloVe | 0.525 | 0.089 | 0.000 | 0.000 | 0.000 | 1.000 | NA | 94 |
|  |  | BioWordVec | 0.561 | 0.100 | 0.046 | 0.083 | 0.005 | 0.994 | 0.087 | 126 |
| Asthma | 13% | No Pre-trained | 0.547 | 0.183 | 0.210 | 0.240 | 0.180 | 0.916 | 0.199 | 197 |
|  |  | GloVe | 0.574 | 0.176 | 0.028 | 0.050 | 0.003 | 0.997 | 0.045 | 71 |
|  |  | BioWordVec | 0.545 | 0.185 | 0.206 | 0.367 | 0.045 | 0.989 | 0.094 | 126 |
| Gout | 13% | No Pre-trained | 0.527 | 0.156 | 0.136 | 0.174 | 0.098 | 0.932 | 0.137 | 192 |
|  |  | GloVe | 0.523 | 0.146 | 0.000 | 0.000 | 0.000 | 0.999 | NA | 76 |
|  |  | BioWordVec | 0.507 | 0.141 | 0.108 | 0.208 | 0.005 | 0.996 | 0.046 | 128 |
| OSA | 14% | No Pre-trained | 0.497 | 0.155 | 0.133 | 0.158 | 0.107 | 0.911 | 0.125 | 193 |
|  |  | GloVe | 0.523 | 0.149 | 0.131 | 0.250 | 0.002 | 0.999 | 0.044 | 79 |
|  |  | BioWordVec | 0.536 | 0.155 | 0.024 | 0.039 | 0.007 | 0.988 | 0.057 | 128 |
| PVD | 15% | No Pre-trained | 0.506 | 0.186 | 0.201 | 0.231 | 0.171 | 0.903 | 0.195 | 197 |
|  |  | GloVe | 0.565 | 0.188 | 0.000 | 0.000 | 0.000 | 0.997 | NA | 93 |
|  |  | BioWordVec | 0.546 | 0.185 | 0.059 | 0.096 | 0.020 | 0.983 | 0.080 | 130 |
| Gallstones | 15% | No Pre-trained | 0.498 | 0.172 | 0.198 | 0.219 | 0.177 | 0.887 | 0.193 | 219 |
|  |  | GloVe | 0.546 | 0.203 | 0.151 | 0.278 | 0.017 | 0.993 | 0.074 | 97 |
|  |  | BioWordVec | 0.537 | 0.207 | 0.196 | 0.348 | 0.040 | 0.986 | 0.097 | 130 |
| OA | 18% | No Pre-trained | 0.482 | 0.179 | 0.133 | 0.162 | 0.104 | 0.883 | 0.125 | 231 |
|  |  | GloVe | 0.545 | 0.207 | 0.127 | 0.250 | 0.002 | 0.998 | 0.035 | 97 |
|  |  | BioWordVec | 0.528 | 0.199 | 0.081 | 0.152 | 0.009 | 0.988 | 0.041 | 127 |
| GERD | 20% | No Pre-trained | 0.502 | 0.214 | 0.166 | 0.212 | 0.121 | 0.883 | 0.152 | 208 |
|  |  | GloVe | 0.504 | 0.210 | NA | NA | 0.000 | 1.000 | NA | 79 |
|  |  | BioWordVec | 0.522 | 0.227 | 0.228 | 0.415 | 0.016 | 0.990 | 0.069 | 130 |
| Depression | 20% | No Pre-trained | 0.515 | 0.215 | 0.216 | 0.240 | 0.192 | 0.845 | 0.211 | 199 |
|  |  | GloVe | 0.568 | 0.252 | 0.149 | 0.285 | 0.011 | 0.992 | 0.042 | 74 |
|  |  | BioWordVec | 0.556 | 0.242 | 0.123 | 0.211 | 0.035 | 0.975 | 0.074 | 131 |
| Obesity | 40% | No Pre-trained | 0.504 | 0.409 | 0.375 | 0.408 | 0.342 | 0.658 | 0.351 | 195 |
|  |  | GloVe | 0.497 | 0.406 | 0.231 | 0.397 | 0.066 | 0.929 | 0.108 | 66 |
|  |  | BioWordVec | 0.501 | 0.422 | 0.257 | 0.429 | 0.086 | 0.925 | 0.140 | 131 |
| CHF | 43% | No Pre-trained | 0.608 | 0.590 | 0.509 | 0.622 | 0.396 | 0.807 | 0.473 | 194 |
|  |  | GloVe | 0.628 | 0.569 | 0.511 | 0.614 | 0.407 | 0.805 | 0.489 | 66 |
|  |  | BioWordVec | 0.637 | 0.578 | 0.487 | 0.617 | 0.357 | 0.830 | 0.449 | 130 |
| Hyper- cholesterolemia | 47% | No Pre-trained | 0.540 | 0.534 | 0.448 | 0.528 | 0.368 | 0.705 | 0.422 | 196 |
|  |  | GloVe | 0.538 | 0.524 | 0.385 | 0.565 | 0.206 | 0.863 | 0.301 | 68 |
|  |  | BioWordVec | 0.535 | 0.520 | 0.393 | 0.570 | 0.216 | 0.860 | 0.306 | 130 |
| CAD | 55% | No Pre-trained | 0.580 | 0.631 | 0.612 | 0.608 | 0.616 | 0.458 | 0.568 | 192 |
|  |  | GloVe | 0.525 | 0.601 | 0.731 | 0.546 | 0.917 | 0.060 | 0.684 | 66 |
|  |  | BioWordVec | 0.523 | 0.593 | 0.711 | 0.546 | 0.877 | 0.103 | 0.673 | 132 |
| Diabetes | 66% | No Pre-trained | 0.608 | 0.754 | 0.719 | 0.716 | 0.721 | 0.394 | 0.698 | 194 |
|  |  | GloVe | 0.571 | 0.730 | 0.831 | 0.665 | 0.997 | 0.003 | 0.798 | 66 |
|  |  | BioWordVec | 0.562 | 0.719 | 0.822 | 0.663 | 0.981 | 0.013 | 0.791 | 133 |
| Hypertension | 73% | No Pre-trained | 0.553 | 0.770 | 0.782 | 0.745 | 0.820 | 0.244 | 0.780 | 191 |
|  |  | GloVe | 0.557 | 0.780 | 0.859 | 0.729 | 0.988 | 0.014 | 0.839 | 66 |
|  |  | BioWordVec | 0.556 | 0.770 | 0.850 | 0.732 | 0.967 | 0.046 | 0.833 | 132 |

**Table 8. Model Performance Evaluation Metrics (Average of 10 Iterations) - LSTM with/without Pre-trained Word Embeddings**

| **Disease** | **Percent Disease** | **Embedding** | **AUC- ROC** | **AUC- PR** | **Balanced**  **Accuracy** | **Precision** | **Recall** | **Specificity** | **F1 Score** | **Running Time (Seconds)** |
| --- | --- | --- | --- | --- | --- | --- | --- | --- | --- | --- |
| Hypertrigly- ceridemia | 5% | No Pre-trained | 0.397 | 0.048 | 0.000 | 0.000 | 0.000 | 0.998 | NA | 187 |
|  |  | GloVe | 0.586 | 0.089 | NA | NA | 0.000 | 1.000 | NA | 84 |
|  |  | BioWordVec | 0.578 | 0.080 | NA | NA | 0.000 | 1.000 | NA | 116 |
| Venous Insufficiency | 7% | No Pre-trained | 0.477 | 0.094 | 0.091 | 0.161 | 0.019 | 0.992 | 0.083 | 175 |
|  |  | GloVe | 0.526 | 0.085 | NA | NA | 0.000 | 1.000 | NA | 88 |
|  |  | BioWordVec | 0.509 | 0.086 | NA | NA | 0.000 | 1.000 | NA | 115 |
| Asthma | 13% | No Pre-trained | 0.479 | 0.154 | 0.151 | 0.245 | 0.058 | 0.971 | 0.100 | 180 |
|  |  | GloVe | 0.570 | 0.169 | 0.000 | 0.000 | 0.000 | 0.997 | NA | 65 |
|  |  | BioWordVec | 0.523 | 0.164 | 0.058 | 0.107 | 0.005 | 0.992 | 0.045 | 116 |
| Gout | 13% | No Pre-trained | 0.513 | 0.140 | 0.013 | 0.021 | 0.005 | 0.980 | 0.039 | 178 |
|  |  | GloVe | 0.521 | 0.138 | 0.000 | 0.000 | 0.000 | 0.997 | NA | 67 |
|  |  | BioWordVec | 0.519 | 0.142 | 0.000 | 0.000 | 0.000 | 0.999 | NA | 118 |
| OSA | 14% | No Pre-trained | 0.500 | 0.155 | 0.120 | 0.186 | 0.053 | 0.961 | 0.089 | 181 |
|  |  | GloVe | 0.520 | 0.146 | NA | NA | 0.000 | 1.000 | NA | 74 |
|  |  | BioWordVec | 0.518 | 0.151 | 0.059 | 0.111 | 0.002 | 0.997 | 0.043 | 118 |
| PVD | 15% | No Pre-trained | 0.442 | 0.151 | 0.118 | 0.190 | 0.047 | 0.972 | 0.092 | 183 |
|  |  | GloVe | 0.543 | 0.185 | 0.180 | 0.327 | 0.013 | 0.995 | 0.080 | 85 |
|  |  | BioWordVec | 0.531 | 0.185 | 0.141 | 0.261 | 0.009 | 0.994 | 0.040 | 120 |
| Gallstones | 15% | No Pre-trained | 0.472 | 0.158 | 0.136 | 0.219 | 0.053 | 0.965 | 0.084 | 199 |
|  |  | GloVe | 0.530 | 0.202 | 0.344 | 0.667 | 0.004 | 0.999 | 0.041 | 86 |
|  |  | BioWordVec | 0.533 | 0.197 | 0.164 | 0.300 | 0.019 | 0.993 | 0.069 | 119 |
| OA | 18% | No Pre-trained | 0.502 | 0.193 | 0.141 | 0.191 | 0.091 | 0.911 | 0.087 | 212 |
|  |  | GloVe | 0.535 | 0.207 | 0.000 | 0.000 | 0.000 | 0.996 | NA | 87 |
|  |  | BioWordVec | 0.533 | 0.203 | 0.077 | 0.132 | 0.011 | 0.982 | 0.053 | 116 |
| GERD | 20% | No Pre-trained | 0.517 | 0.229 | 0.120 | 0.206 | 0.034 | 0.973 | 0.072 | 185 |
|  |  | GloVe | 0.534 | 0.229 | NA | NA | 0.000 | 1.000 | NA | 72 |
|  |  | BioWordVec | 0.504 | 0.214 | 0.191 | 0.363 | 0.008 | 0.993 | 0.048 | 118 |
| Depression | 20% | No Pre-trained | 0.504 | 0.221 | 0.168 | 0.245 | 0.092 | 0.933 | 0.129 | 184 |
|  |  | GloVe | 0.540 | 0.239 | 0.058 | 0.096 | 0.008 | 0.995 | 0.133 | 67 |
|  |  | BioWordVec | 0.540 | 0.239 | 0.154 | 0.247 | 0.048 | 0.970 | 0.105 | 120 |
| Obesity | 40% | No Pre-trained | 0.492 | 0.407 | 0.274 | 0.402 | 0.146 | 0.854 | 0.211 | 181 |
|  |  | GloVe | 0.507 | 0.413 | 0.219 | 0.379 | 0.059 | 0.935 | 0.101 | 62 |
|  |  | BioWordVec | 0.516 | 0.420 | 0.298 | 0.457 | 0.138 | 0.886 | 0.205 | 120 |
| CHF | 43% | No Pre-trained | 0.655 | 0.623 | 0.547 | 0.638 | 0.455 | 0.792 | 0.518 | 179 |
|  |  | GloVe | 0.648 | 0.575 | 0.502 | 0.602 | 0.402 | 0.798 | 0.476 | 60 |
|  |  | BioWordVec | 0.641 | 0.586 | 0.495 | 0.637 | 0.354 | 0.840 | 0.448 | 120 |
| Hyper- cholesterolemia | 47% | No Pre-trained | 0.536 | 0.526 | 0.422 | 0.534 | 0.311 | 0.756 | 0.372 | 179 |
|  |  | GloVe | 0.545 | 0.522 | 0.400 | 0.576 | 0.223 | 0.858 | 0.320 | 61 |
|  |  | BioWordVec | 0.532 | 0.512 | 0.388 | 0.553 | 0.224 | 0.842 | 0.316 | 121 |
| CAD | 55% | No Pre-trained | 0.561 | 0.623 | 0.634 | 0.590 | 0.678 | 0.392 | 0.597 | 176 |
|  |  | GloVe | 0.525 | 0.604 | 0.723 | 0.549 | 0.898 | 0.091 | 0.681 | 61 |
|  |  | BioWordVec | 0.536 | 0.606 | 0.707 | 0.550 | 0.865 | 0.131 | 0.671 | 121 |
| Diabetes | 66% | No Pre-trained | 0.600 | 0.746 | 0.777 | 0.678 | 0.877 | 0.154 | 0.752 | 178 |
|  |  | GloVe | 0.589 | 0.735 | 0.831 | 0.665 | 0.996 | 0.008 | 0.798 | 60 |
|  |  | BioWordVec | 0.591 | 0.749 | 0.818 | 0.665 | 0.971 | 0.033 | 0.789 | 124 |
| Hypertension | 73% | No Pre-trained | 0.543 | 0.763 | 0.804 | 0.736 | 0.873 | 0.148 | 0.781 | 176 |
|  |  | GloVe | 0.545 | 0.773 | 0.857 | 0.732 | 0.982 | 0.035 | 0.839 | 61 |
|  |  | BioWordVec | 0.547 | 0.766 | 0.842 | 0.731 | 0.953 | 0.055 | 0.827 | 125 |

**Table 9. Model Performance Evaluation Metrics (Average of 10 Iterations) - Bi-LSTM with/without Pre-trained Word Embeddings**

| **Disease** | **Percent Disease** | **Embedding** | **AUC- ROC** | **AUC- PR** | **Balanced**  **Accuracy** | **Precision** | **Recall** | **Specificity** | **F1 Score** | **Running Time (Seconds)** |
| --- | --- | --- | --- | --- | --- | --- | --- | --- | --- | --- |
| Hypertrigly- ceridemia | 5% | No Pre-trained | 0.481 | 0.091 | 0.093 | 0.144 | 0.041 | 0.986 | 0.121 | 266 |
|  |  | GloVe | 0.569 | 0.073 | NA | NA | 0.000 | 1.000 | NA | 121 |
|  |  | BioWordVec | 0.528 | 0.076 | NA | NA | 0.000 | 1.000 | NA | 150 |
| Venous Insufficiency | 7% | No Pre-trained | 0.558 | 0.140 | 0.180 | 0.280 | 0.071 | 0.988 | 0.138 | 252 |
|  |  | GloVe | 0.586 | 0.097 | NA | NA | 0.000 | 1.000 | NA | 124 |
|  |  | BioWordVec | 0.590 | 0.096 | 0.000 | 0.000 | 0.000 | 0.998 | NA | 147 |
| Asthma | 13% | No Pre-trained | 0.589 | 0.232 | 0.276 | 0.423 | 0.130 | 0.974 | 0.196 | 276 |
|  |  | GloVe | 0.622 | 0.199 | 0.263 | 0.500 | 0.008 | 0.998 | 0.047 | 91 |
|  |  | BioWordVec | 0.606 | 0.211 | 0.156 | 0.271 | 0.033 | 0.991 | 0.111 | 161 |
| Gout | 13% | No Pre-trained | 0.643 | 0.300 | 0.300 | 0.417 | 0.183 | 0.960 | 0.248 | 255 |
|  |  | GloVe | 0.652 | 0.224 | 0.268 | 0.500 | 0.007 | 1.000 | 0.136 | 94 |
|  |  | BioWordVec | 0.773 | 0.452 | 0.460 | 0.707 | 0.193 | 0.984 | 0.299 | 154 |
| OSA | 14% | No Pre-trained | 0.647 | 0.292 | 0.314 | 0.412 | 0.216 | 0.943 | 0.270 | 251 |
|  |  | GloVe | 0.623 | 0.198 | 0.045 | 0.067 | 0.005 | 0.995 | 0.069 | 106 |
|  |  | BioWordVec | 0.710 | 0.298 | 0.243 | 0.398 | 0.088 | 0.979 | 0.175 | 162 |
| PVD | 15% | No Pre-trained | 0.635 | 0.349 | 0.377 | 0.511 | 0.242 | 0.960 | 0.327 | 251 |
|  |  | GloVe | 0.624 | 0.234 | 0.052 | 0.100 | 0.002 | 0.998 | 0.043 | 123 |
|  |  | BioWordVec | 0.685 | 0.342 | 0.361 | 0.555 | 0.167 | 0.974 | 0.244 | 167 |
| Gallstones | 15% | No Pre-trained | 0.512 | 0.190 | 0.189 | 0.280 | 0.098 | 0.950 | 0.138 | 289 |
|  |  | GloVe | 0.561 | 0.206 | 0.195 | 0.367 | 0.019 | 0.995 | 0.069 | 125 |
|  |  | BioWordVec | 0.575 | 0.219 | 0.148 | 0.254 | 0.043 | 0.982 | 0.090 | 171 |
| OA | 18% | No Pre-trained | 0.593 | 0.330 | 0.345 | 0.432 | 0.257 | 0.917 | 0.315 | 290 |
|  |  | GloVe | 0.594 | 0.272 | 0.269 | 0.512 | 0.018 | 0.994 | 0.067 | 128 |
|  |  | BioWordVec | 0.648 | 0.344 | 0.321 | 0.518 | 0.125 | 0.977 | 0.220 | 153 |
| GERD | 20% | No Pre-trained | 0.544 | 0.233 | 0.207 | 0.257 | 0.158 | 0.887 | 0.193 | 264 |
|  |  | GloVe | 0.557 | 0.253 | 0.103 | 0.200 | 0.003 | 0.998 | 0.031 | 104 |
|  |  | BioWordVec | 0.540 | 0.226 | 0.105 | 0.182 | 0.023 | 0.979 | 0.055 | 164 |
| Depression | 20% | No Pre-trained | 0.608 | 0.296 | 0.284 | 0.379 | 0.190 | 0.912 | 0.244 | 260 |
|  |  | GloVe | 0.616 | 0.270 | 0.163 | 0.307 | 0.015 | 0.990 | 0.044 | 96 |
|  |  | BioWordVec | 0.616 | 0.271 | 0.187 | 0.313 | 0.061 | 0.960 | 0.103 | 167 |
| Obesity | 40% | No Pre-trained | 0.610 | 0.526 | 0.486 | 0.537 | 0.435 | 0.744 | 0.478 | 258 |
|  |  | GloVe | 0.624 | 0.539 | 0.448 | 0.553 | 0.343 | 0.808 | 0.414 | 87 |
|  |  | BioWordVec | 0.672 | 0.593 | 0.553 | 0.587 | 0.518 | 0.750 | 0.540 | 168 |
| CHF | 43% | No Pre-trained | 0.781 | 0.714 | 0.679 | 0.690 | 0.668 | 0.767 | 0.677 | 258 |
|  |  | GloVe | 0.779 | 0.716 | 0.670 | 0.697 | 0.643 | 0.784 | 0.668 | 85 |
|  |  | BioWordVec | 0.821 | 0.765 | 0.702 | 0.721 | 0.684 | 0.803 | 0.696 | 173 |
| Hyper- cholesterolemia | 47% | No Pre-trained | 0.635 | 0.568 | 0.574 | 0.562 | 0.587 | 0.601 | 0.572 | 270 |
|  |  | GloVe | 0.620 | 0.565 | 0.544 | 0.572 | 0.516 | 0.660 | 0.536 | 87 |
|  |  | BioWordVec | 0.623 | 0.570 | 0.544 | 0.563 | 0.526 | 0.641 | 0.537 | 174 |
| CAD | 55% | No Pre-trained | 0.780 | 0.790 | 0.763 | 0.739 | 0.786 | 0.658 | 0.762 | 254 |
|  |  | GloVe | 0.781 | 0.816 | 0.734 | 0.743 | 0.725 | 0.685 | 0.731 | 85 |
|  |  | BioWordVec | 0.874 | 0.872 | 0.839 | 0.813 | 0.864 | 0.745 | 0.834 | 177 |
| Diabetes | 66% | No Pre-trained | 0.724 | 0.819 | 0.806 | 0.760 | 0.853 | 0.461 | 0.803 | 253 |
|  |  | GloVe | 0.671 | 0.779 | 0.786 | 0.721 | 0.851 | 0.343 | 0.779 | 85 |
|  |  | BioWordVec | 0.890 | 0.930 | 0.880 | 0.871 | 0.888 | 0.727 | 0.877 | 182 |
| Hypertension | 73% | No Pre-trained | 0.574 | 0.762 | 0.807 | 0.758 | 0.856 | 0.267 | 0.804 | 256 |
|  |  | GloVe | 0.610 | 0.784 | 0.849 | 0.734 | 0.963 | 0.062 | 0.833 | 86 |
|  |  | BioWordVec | 0.617 | 0.791 | 0.820 | 0.761 | 0.879 | 0.257 | 0.815 | 182 |
